# Adult height, coronary heart disease and stroke: a multi-locus Mendelian randomization meta-analysis

**DOI:** 10.1093/ije/dyv074

**Published:** 2015-05-15

**Authors:** Eveline Nüesch, Caroline Dale, Tom M Palmer, Jon White, Brendan J Keating, Erik PA van Iperen, Anuj Goel, Sandosh Padmanabhan, Folkert W Asselbergs, EPIC-Netherland Investigators, WM Verschuren, C Wijmenga, YT Van der Schouw, NC Onland-Moret, Leslie A Lange, GK Hovingh, Suthesh Sivapalaratnam, Richard W Morris, Peter H Whincup, Goya S Wannamethe, Tom R Gaunt, Shah Ebrahim, Laura Steel, Nikhil Nair, Alexander P Reiner, Charles Kooperberg, James F Wilson, Jennifer L Bolton, Stela McLachlan, Jacqueline F Price, Mark WJ Strachan, Christine M Robertson, Marcus E Kleber, Graciela Delgado, Winfried März, Olle Melander, Anna F Dominiczak, Martin Farrall, Hugh Watkins, Maarten Leusink, Anke H Maitland-van der Zee, Mark CH de Groot, Frank Dudbridge, Aroon Hingorani, Yoav Ben-Shlomo, Debbie A Lawlor, UCLEB Investigators, A Amuzu, M Caufield, A Cavadino, J Cooper, TL Davies, IN Day, F Drenos, J Engmann, C Finan, C Giambartolomei, R Hardy, SE Humphries, E Hypponen, M Kivimaki, D Kuh, M Kumari, K Ong, V Plagnol, C Power, M Richards, S Shah, T Shah, R Sofat, PJ Talmud, N Wareham, H Warren, JC Whittaker, A Wong, D Zabaneh, George Davey Smith, Jonathan C Wells, David A Leon, Michael V Holmes, Juan P Casas

**Affiliations:** 1 Faculty of Epidemiology and Population Health, London School of Hygiene and Tropical Medicine, London, UK; 2 CTU Bern, Department of Clinical Research and Institute of Social and Preventive Medicine (ISPM), University of Bern, Bern, Switzerland; 3 Warwick Medical School, University of Warwick, Coventry, UK; 4 Department of Mathematics and Statistics, Lancaster University, Lancaster, UK; 5 UCL Genetics Institute, Department of Genetics, Evolution and Environment, University College London, London, UK; 6 Department of Pediatrics, Perelman School of Medicine, University of Pennsylvania, Philadelphia, PA, USA; 7 Department of Surgery; 8 Division of Genetics, University of Pennsylvania, Philadelphia; 9 Department of Biostatistics, Academic Medical Center Amsterdam, Amsterdam, The Netherlands; 10 Wellcome Trust Centre for Human Genetics and Radcliffe Department of Medicine, University of Oxford, Oxford, UK; 11 Institute of Cardiovascular and Medical Sciences, College of Medical Veterinary and Life Sciences, University of Glasgow, Glasgow, UK; 12 Department of Cardiology, Division Heart and Lungs, University Medical Centre Utrecht, Utrecht, The Netherlands; 13 Durrer Center for Cardiogenetic Research, ICIN-Netherlands Heart Institute, Utrecht, The Netherlands; 14 Institute of Cardiovascular Science, Faculty of Population Health Sciences, University College London, London, UK; 15 EPIC-NL, Bilthoven and Utrecht, The Netherlands; 16 Department of Genetics, University of North Carolina at Chapel Hill, Chapel Hill, NC, USA; 17 Department of Vascular Medicine, Academic Medical Center Amsterdam, Amsterdam, The Netherlands; 18 Department of Primary Care & Population Health, University College London, London, UK; 19 Population Health Research Institute, St George's, University of London, London, UK; 20 MRC Integrative Epidemiology Unit, School of Social and Community Medicine, University of Bristol, Bristol, UK; 21 School of Social and Community Medicine, University of Bristol, Bristol, UK; 22 Department of Epidemiology, University of Washington, Seattle, WA, USA / Division of Public Health Sciences, Fred Hutchinson Cancer Research Center, Seattle, WA, USA; 23 Division of Public Health Sciences, Fred Hutchinson Cancer Research Center, Seattle, WA, USA; 24 Usher Institute for Population Health Sciences and Informatics, University of Edinburgh, Edinburgh, UK; 25 MRC Human Genetics Unit, Institute of Genetics and Molecular Medicine, University of Edinburgh, Edinburgh, UK; 26 Metabolic Unit, Western General Hospital, Edinburgh, UK; 27 Fifth Department of Medicine, Medical Faculty Mannheim, Heidelberg University, Heidelberg, Germany; 28 Medical Clinic V (Nephrology, Hypertensiology, Endocrinology, Diabetolgy, and Rheumatology), Mannheim Medical Faculty, University of Heidelberg, Germany, Synlab Academy, Synlab Services GmbH, Mannheim and Augsburg, Germany, Clinical Institute of Medical and Chemical Laboratory Diagnostics, Medical University of Graz, Austria; 29 Lund University, Sweden; 30 Division of Pharmacoepidemiology & Clinical Pharmacology, Utrecht Institute for Pharmaceutical Sciences, Utrecht University, Utrecht, The Netherlands; 31 Julius Center for Health Sciences and Primary Care, University Medical Center Utrecht, Utrecht, The Netherlands; 32 Department of Epidemiology and Public Health, University College London Medical School, London, UK; 33 UCLEB, London, Edinburgh and Bristol, UK; 34 Childhood Nutrition Research Centre, UCL Institute of Child Health, London, UK; 35 Department of Community Medicine, Arctic University of Norway, UiT; 36 Department of Surgery and Clinical Epidemiology Unit, Center for Clinical Epidemiology and Biostatistics, Perelman School of Medicine, University of Pennsylvania, Philadelphia, PA, USA; 37 Clinical Trial Service Unit & Epidemiological Studies Unit (CTSU), Nuffield Department of Population Health, University of Oxford, Oxford, UK

## Abstract

**Background:** We investigated causal effect of completed growth, measured by adult height, on coronary heart disease (CHD), stroke and cardiovascular traits, using instrumental variable (IV) Mendelian randomization meta-analysis.

**Methods:** We developed an allele score based on 69 single nucleotide polymorphisms (SNPs) associated with adult height, identified by the IBCCardioChip, and used it for IV analysis against cardiovascular risk factors and events in 21 studies and 60 028 participants. IV analysis on CHD was supplemented by summary data from 180 height-SNPs from the GIANT consortium and their corresponding CHD estimates derived from CARDIoGRAMplusC4D.

**Results:** IV estimates from IBCCardioChip and GIANT-CARDIoGRAMplusC4D showed that a 6.5-cm increase in height reduced the odds of CHD by 10% [odds ratios 0.90; 95% confidence intervals (CIs): 0.78 to 1.03 and 0.85 to 0.95, respectively],which agrees with the estimate from the Emerging Risk Factors Collaboration (hazard ratio 0.93; 95% CI: 0.91 to 0.94). IV analysis revealed no association with stroke (odds ratio 0.97; 95% CI: 0.79 to 1.19). IV analysis showed that a 6.5-cm increase in height resulted in lower levels of body mass index (*P* < 0.001), triglycerides (*P* < 0.001), non high-density (non-HDL) cholesterol (*P* < 0.001), C-reactive protein (*P* = 0.042), and systolic blood pressure (*P* = 0.064) and higher levels of forced expiratory volume in 1 s and forced vital capacity (*P* < 0.001 for both).

**Conclusions:** Taller individuals have a lower risk of CHD with potential explanations being that taller people have a better lung function and lower levels of body mass index, cholesterol and blood pressure.

## Introduction

Observational studies have shown associations of adult height used as a measure of completed growth, with major non-communicable diseases.^1^^,^^2^^,^^3^^,^^4^ Studying over 1 million participants, the Emerging Risk Factors Collaboration (ERFC) found a 6% decrease in risk of dying from coronary heart disease (CHD) and stroke per 6.5 cm increase in adult height.^4^ Controversy remains about the explanations for these associations. Some authors suggest adult height is only a proxy of circumstances affecting growth in infancy and childhood,^2^^,^^5^ whereas others argue for confounding by behavioural, psychosocial and biological factors. Finally, reverse causation could arise from ‘shrinkage’ in early stages of disease.^1^^,^^5^^,^^6^

Given that genetic variants are unlikely to be affected by the wide range of confounders that usually bias multivariable analyses and cannot be influenced by reverse causality, we employed a multiple instruments Mendelian randomization approach^7^^,^^8^^,^^9^ to investigate the causal effect of completed growth, measured by adult height, with CHD and stroke and examine several cardiovascular traits to gain insight about potential mechanisms.

## Methods

We included individual participant data from 60 028 participants of European ancestry from 21 prospective studies (for details see Table S1, available as Supplementary data at *IJE* online) with recorded standing adult height and at least one of the outcomes (CHD or stroke). All participating studies obtained informed consent for DNA analysis and received ethical approval.

Two multiple instruments were created. The first incorporates 69 loci identified in a gene-centric meta-analysis of height with the Institute for Translational Medicine and Therapeutics (ITMAT) Broad Institute CARe consortium (IBC) CardioChip array^10^^,^^11^ (a chip designed to assess SNPs across relevant loci for a range of cardiovascular disease (CVD) syndromes), and was applied in 21 prospective studies (60 028 participants) with access to individual participant data. The second was based on summary data from 180 statistically independent height-associated SNPs from the Genetic Investigation of ANthropometric Traits (GIANT) Consortium^12^ and their corresponding summary CHD estimates derived from the Coronary Artery Disease Genome-wide Replication and Meta-analysis (CARDIoGRAM) plus the Coronary Artery Disease (C4D) Genetics Consorium, collectively known as CARDIoGRAMplusC4D,^13^ downloaded from [http://www.CARDIOGRAMPLUSC4D.org]. The GIANT Consortium^12^ was a meta-genome-wide association study (GWAs) including 183 727 individuals, that identified 180 independent loci associated with adult height, explaining 10% of the phenotypic variation. The CARDIoGRAMplusC4D Consortium^13^ identified SNPs associating with CHD in 63 746 CAD cases and 130 681 controls.

Genotyping was conducted with the IBC Cardiochip array in 16 studies,^14^ and with Metabochip^15^ in the five remaining studies (Table S2, available as Supplementary data at *IJE* online).We selected 69 SNPs, representing 69 different loci, independently associated with adult height at array-wide significance (*P* < 2.4×10^−6^) in gene-centric meta-analysis of height from 114 223 individuals and 47 studies genotyped with the IBC Cardiochip array (including 16 studies also analysed here) to construct the allele score.^10^ In the five studies with Metabochip genotyping, we used imputed SNPs in linkage disequilibrium (R^2 ^> 0.8) with those from IBC Cardiochip.^16^ SNPs were coded as 0, 1 and 2 indicating the number of height-raising alleles. A per-allele positive effect weighted by the summary beta coefficients from the meta-analysis was summed for each risk allele to construct an allele score.^10^ In a sensitivity analysis, allele scores were constructed without weighting, to address potential overfitting given that the studies included here contributed to the meta-analysis that provided the weights.

The primary outcome was prevalent or incident (fatal and non-fatal) CHD. The secondary outcome was prevalent or incident (fatal and non-fatal) stroke including haemorrhagic or ischaemic events. Validated events were preferred over non-validated, self-reported events. Details for outcome definitions in each study are provided in Table S3, available as Supplementary data at *IJE* online.

To gain insight into the mechanisms that may explain the association of height with CHD and stroke, we used available information from individual studies on established or promising risk factors for CHD and stroke [sex, age, blood pressure, body mass index, smoking, type 2 diabetes, high-density lipoprotein (HDL) cholesterol, non-HDL cholesterol, triglycerides, fasting glucose, C-reactive protein; for details see Table S4, available as Supplementary data at *IJE* online] and on lung function [forced expiratory volume in 1 s (FEV1), forced vital capacity (FVC)] given the established association with adult height.^2^

### Statistical analysis

The same analytical script was used by all studies. For each of the SNPs (69 SNPs for studies using IBC CardioChip and 35 for studies using Metabochip; Table S5, available as Supplementary data at *IJE* online), we calculated frequencies of the height-increasing allele and *P*-values for Hardy–Weinberg equilibrium. In each study, we fitted regression models to estimate the association between adult height and the allele score, with the allele score treated as a continuous trait or divided into deciles. We estimated the proportion of variance (R^2^) of height explained by the allele score and the corresponding standard error by bootstrapping. We used inverse-variance weighted fixed-effects meta-analysis to pool estimates across studies.^17^

We used linear or logistic regression models to examine the genetic association of the allele score with clinical events, cardiovascular traits and confounders (smoking) in individual studies. Owing to skewed distributions, triglycerides and C-reactive protein were analysed on the natural logarithmic scale. For comparability across cardiovascular traits, the original values were divided by the standard deviation. Fixed-effect meta-analysis was used to estimate pooled associations across studies.

For the instrumental variable (IV) analysis, we used the logistic control function estimator to estimate study-specific odds ratios (ORs) between height and clinical events.^18^^,^^19^ This involved a two-stage process: we first conducted within each study a linear regression analysis with adult height as the dependent variable and the allele score as the independent variable. The residuals from the first step were then incorporated into a logistic regression model of the binary trait on the predicted adult height from the first stage. We specified heteroskedasticity robust standard errors in the second stage to incorporate the uncertainty in the estimated residuals from the first stage. Results were expressed as odds ratios (ORs) per 6.5 cm height with corresponding 95% confidence intervals (CIs) to make them comparable to observational estimates from the ERFC.^4^ For continuous traits, we used two-stage least squares analysis using the allele score as the IV for adult height. We also fitted IV models including the following cardiovascular risk factors as covariables: systolic blood pressure, body mass index, lipids [triglycerides, non-HDL cholesterol], lung function [FEV1, FVC] and C-reactive protein. The reason we selected these traits is that they were identified (by our genetic instrument) as potential downstream biological consequences of height. This approach required that studies had measured the traits of interest; studies without this information were excluded.

We pooled study-specific instrumental variable estimates using fixed-effects meta-analysis.^20^ We calculated I^2^ statistics to quantify heterogeneity between studies and derived *P*-values from Cochran’s Q test.^21^ All *P*-values are two-sided.

In a separate analysis, we used published data from both the GIANT and CARDIoGRAMplusC4D consortia to conduct a multiple instrument Mendelian randomization meta-analysis of adult height on CHD using summary level data. For the 180 GWAs height loci reported in GIANT, we extracted the rs number, beta coefficient, effect allele and *P*-value. We approximated the Z statistic by taking the inverse cumulative standard normal distribution of the *P*-value and divided the beta coefficient by the Z statistic to obtain the standard error. We identified the corresponding SNPs and summary estimates for CHD in the CARDIoGRAMplusC4D Consortium and arranged SNPs so that the estimates for height and CHD corresponded to the same reference allele (Table S6, available as Supplementary data at *IJE* online). Using the summary estimates for height and CHD, we synthesized instrumental variable estimates for each SNP by dividing the SNP-CHD association by the SNP-height association and using the delta method to approximate the standard error.^22^ This generated an instrumental variable estimate for each of the 180 individual SNPs, which we pooled using fixed-effects meta-analysis to yield a summary effect of height on CHD.^20^

## Results

A total of 21 studies (60 028 participants) with data on IBC CardioChip array were included (Tables S2 and S4) with a median age at baseline of 61 years (range 26 to 74 years), and 51% were women (range 0% to 100%). The median height was 169 cm (range 156 to 175 cm). In total, there were10 848 CHD and 4 878 stroke cases.

**Figure 1.**
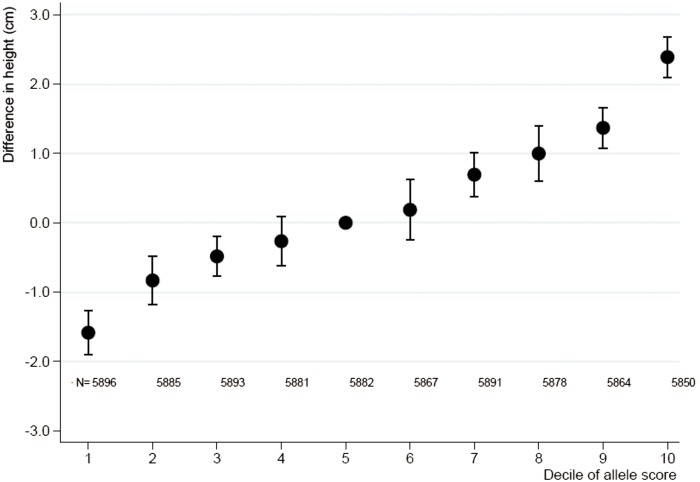
Meta-analysis pooled estimates for the association between deciles of the allele score and adult height. Presented are pooled differences in mean adult height with corresponding 95% confidence intervals as compared with the 5th decile, derived from fixed-effect meta-analysis. N, numbers analysed in each decile.

Adult height increased by 0.79 cm (95% CI: 0.75 cm, 0.84 cm) per one-unit increase in allele score derived from the IBC CardioChip array with low heterogeneity across studies (I^2 ^= 29%, *P* = 0.108) (Figure 1), and explained 1.4% (95% CI: 1.2%, 1.5%) of the variance in adult height. The allele score showed no association with smoking (20 studies with 57 075 participants including 32 665 smokers, OR 0.97; 95% CI: 0.87, 1.07).

**Figure 2.**
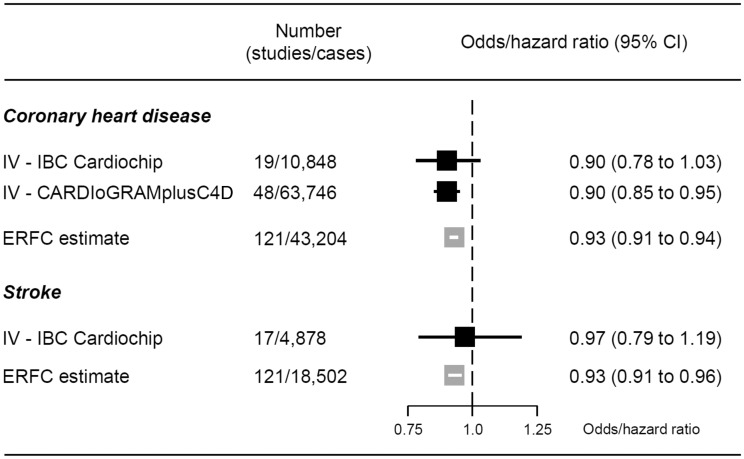
Meta-analysis pooled causal effects for a 6.5-cm increase in adult height on the risk of cardiovascular disease. Odds ratios and corresponding 95% confidence intervals (CI) are estimated from fixed-effect meta-analysis of instrumental variable (IV) estimates from individual studies. Hazard ratios are taken from estimates published by the Emerging Risk Factors Collaboration (ERFC).^4^ Effect estimates are per 6.5 cm increase in adult height. An estimate below 1 indicates that increasing adult height decreases the risk of cardiovascular events.

An IV analysis, using the allele score derived from IBC CardioChip array, that included 19 studies with 10 848 prevalent or incident CHD cases, found that for each 6.5-cm increase in adult height the pooled OR of CHD was 0.90 (95% CI: 0.78, 1.03). The corresponding IV estimate derived from summary data from 180 independent SNPs from the GIANT and CARDIoGRAMplusC4D Consortia (including up to 183 727 individuals with height and 63 746 CHD cases) yielded an OR of CHD of 0.90 (95% CI: 0.85, 0.95) for the same difference in adult height. These IV estimates were in agreement with the observational estimate reported by the ERFC (hazard ratio 0.93, 95% CI: 0.91, 0.94, Figure 2).^4^

We analysed 4 878 cases of stroke in 43 790 participants in 17 studies (Figure 2) with data on IBC CardioChip array. The ERFC observed an OR of 0.93 (95% CI: 0.91, 0.96) per 6.5 cm difference in adult height. Our pooled IV estimate for the same height difference showed a not very dissimilar point estimate, though with wide confidence intervals (OR 0.97, 95% CI: 0.79, 1.19).

Study-specific causal estimates for the effect of height on risk of CHD and stroke in the studies for which we had access to participant data are presented in Figures S1 and S2 (available as Supplementary data at *IJE* online). These did not show a relationship between study precision and the IV estimate, which would arise from weak instrument bias and thus bias the overall meta-analysis of IV estimates. SNP-specific instrumental variable estimates derived from summary-level data (GIANT and CARDIoGRAMplusC4) are presented in Figure S3 and a cross-hair plot showing the relationship of height and risk of CHD across the SNPs is presented in Figure S4 (available as Supplementary data at *IJE* online). These show significant heterogeneity, suggesting that the causal effect identified by the allele score is a composite of multiple causal pathways which identify different magnitudes of causal effect.

Instrumental variable analyses of height derived from IBC CardioChip array on cardiovascular traits, showed that an increase of 6.5 cm in adult height had the strongest association with lung function, with a difference of 0.26 standard deviation (SD) units of FEV1 (95% CI: 0.15, 0.36) and of 0.30 SD units of FVC (95% CI: 0.20, 0.41) (Table 1). An increase of 6.5 cm in height associated with lower levels of body mass index (−0.10 SD units, 95% CI: -0.15, −0.05), triglycerides (−0.10 SD, −0.16, −0.05), non-HDL cholesterol (−0.12 SD, −0.17, −0.06), C-reactive protein (−0.07 SD, −0.13, −0.00) and a trend to lower levels in systolic blood pressure (−0.05 SD, −0.10, 0.00). We did not find evidence for an association between adult height and fasting glucose (−0.04 SD, −0.10, 0.01) or type 2 diabetes (19 studies, 60 171 participants,7340 cases, OR per 6.5 cm height 0.99, 95% CI: 0.85, 1.15).

**Table 1.** Meta-analysis pooled estimates derived from instrumental variable analysis for a 6.5-cm increase in adult height on cardiovascular traits. Traits are sorted according to the magnitude of the association observed with adult height

Characteristic	No studies/ participants	Difference per SD in trait for a 6.5-cm increase in height (95% CI)	*P* value, Z-test	Heterogeneity, *I*^2^ (Cochran’s Q test *P*-value)
FVC	6/11129	0.30 (0.20, 0.41)	<0.001	0% (0.774)
FEV_1_	6/11131	0.26 (0.15, 0.36)	<0.001	0% (0.735)
Non-HDL cholesterol	17/41477	–0.12 (–0.17, –0.06)	<0.001	32% (0.102)
Triglycerides^a^	17/42117	–0.10 (–0.16, –0.05)	<0.001	0% (0.513)
Body mass index	20/54099	–0.10 (–0.15, –0.05)	<0.001	30% (0.100)
C-reactive protein^a^	15/35538	–0.07 (–0.13, –0.00)	0.042	0% (0.761)
Systolic blood pressure	19/52345	–0.05 (0.10, 0.00)	0.064	0% (0.467)
Fasting glucose	18/40451	–0.04 (–0.10, 0.01)	0.127	13% (0.302)
HDL cholesterol	18/43030	0.02 (–0.03, 0.08)	0.432	33% (0.089)

Results from instrumental variable analyses are derived from 2-stage least-squares regression and pooled using fixed-effects meta-analysis.

FEV_1_, forced expiratory volume in 1 s; FVC, forced vital capacity; HDL, high-density lipoprotein.

^a^Effects of triglycerides and C-reactive protein are estimated after log-transformation. A negative difference indicates that levels of traits decrease with an increase in adult height.

In an exploratory analysis using a sub-sample of prospective studies with clinical events and cardiovascular traits, we conducted a multivariate IV analysis that included height and the traits that showed an association with the gene score for height (blood pressure, BMI, lipids, lung function and CRP). These analyses suggested a diminution of the IV estimate between adult height and CHD (Figure S5, available as Supplementary data at *IJE* online). The null effect for stroke remained unchanged after adjustment for these traits.

Results for IV analysis derived from the IBC CardioChip array with CHD, stroke and cardiovascular traits using an unweighted allele score were similar to those externally weighted (Table S7, available as Supplementary data at *IJE* online).

## Discussion

In order to investigate the causal effect of completed growth, measured by adult height, on cardiovascular events and traits, we used multiple genetic instruments (derived from IBC CardioChip array, and GIANT-CARDIoGRAMplusC4D consortia) associated with adult height representing completed growth, and through IV analysis showed that genetically taller individuals had a lower risk of CHD as well as differences in several cardiovascular (CV) traits that may explain the cardio-protective effect. The IV estimate indicates a 10% risk reduction in CHD for every 6.5 cm increase in standing height. This is in agreement with the observational estimate reported by the ERFC.^4^ Although we did not find evidence for a causal effect between adult height and risk of stroke, the IV estimate was imprecise, meaning we cannot exclude a causal effect.

Our analysis showed that genetically taller individuals had lower levels of adiposity (body mass index), lipid fractions (non-HDL cholesterol and triglycerides) and better lung function. Our results suggest that these physiological variables may contribute to explain the association of adult height (and completed growth) on CHD. Additional biological mechanisms, which we were not able to explore, might also explain the observed effects on CHD. For example, shorter individuals have smaller vessel calibre,^23^ which becomes more easily occluded leading to increased arterial occlusive events,^24^ and have a higher risk of more advanced coronary atherosclerosis.^25^ Shorter individuals also have faster heart rate and increased augmentation of the primary systolic pulse, indicating greater ventricular systolic work.^26^

According to the principles of Mendelian randomization, we would expect the genetic variants to be evenly distributed with respect to potential confounding factors. However in this particular case, with respect to exposures acting in utero, a potential confounding factor described as a “dynastic effect” may lead to an imbalance whereby adults carrying more height raising alleles may have experienced greater on average maternal height (due to genomic sharing between mothers and offsping). This greater maternal height could affect the in utero environment experienced by the offspring, which could possibly influence their long-term health.^27^ However, these early environmental determinants of adult height are unlikely to entirely explain the association of adult height with CHD, derived from our IV analysis. However, it does not mean that early growth, a key period when these genes act, is not an important mechanism.^28^ Since genotype is invariant, our results also suggest that the reverse causation phenomenon of ‘shrinkage’ due to illness does not explain our effect of adult height on CHD. Furthermore, we did not observe an association between the allele score and smoking, which is a potential confounder.

Mendelian randomization studies using IV methods have been used for causal inference for a broad range of environmental exposures and diseases.^29^^,^^30^^,^^31^ However, the validity of the IV results depends on whether or not the IV assumptions (strength of the genetic instrument, minimization of confounding and specificity) hold in each specific case. First, we used multiple genetic instruments that substantially increase the proportion of the variance in height explained by the instrument (1.4% by IBC CardioChip array and 10% by GIANT consortium), which together with a large number of clinical events (in particular for CHD) lead us to counteract weak instrument bias. Of note, there was no evidence that smaller studies were more affected by weak instrument bias. Second, our genetic instrument for height was not associated with smoking, showing the ability to reduce confounding due to Mendel’s second law. Third, the use of multiple instruments increased the specificity of our genetic instrument (compared with any single instrument);^18^ this is especially important for non-protein traits that are not encoded for by a specific gene. Thus, although the significant heterogeneity among individual SNP IV estimates suggests possible non-specificity for some SNPs, taken together we expect the multiple instruments to have greater specificity, reflected in the similar IV estimates from the 69 and 180 SNP instruments. As a consequence of the strength and specificity of our genetic instrument, we were also able to dissect the downstream biological consequence of the intermediate trait of interest (i.e. differences in blood pressure, lung function and lipid traits as consequence of differences in adult height), known as vertical pleiotropy^32^^,^^33^ and its presence does not violate the IV assumptions. We weighted the IBC CardioChip allele score by the summary beta coefficients from the meta-analysis,^10^ which included all of the studies in this analysis and, therefore, there is a risk of overfitting in the IV estimates. However, when we compared our results with IV results obtained from unweighted scores, we found very similar results,^9^^,^^34^^,^^35^ suggesting that the magnitude of potential overfitting to be unlikely to invalidate our findings. Fourth, our findings are in alignment with a very recent Mendelian randomization study of height and risk of coronary artery disease;^36^ we additionally considered the effect of height on risk of stroke, and our access to participant data from the UCLEB consortium allowed us to explore potential mediators of the relationship between adult height and CHD.

An interesting finding from our IV analysis on cardiovascular traits was that taller people tend to have lower body mass index. Previous studies have shown that whereas adult height is directly associated with most body girths, it is inversely associated with waist girth.^37^ A link between short stature and adiposity may emerge through associations between poor growth in early life and altered metabolism,^38^ which may allow partial reduction of the height deficit while also favouring insulin resistance and central fat accumulation.

The main strength of our study is use of the Mendelian randomization approach incorporating data from two very large consortia consisting of studies with validated cardiovascular endpoints, and two different multiple-genetic instruments. Our approach of using multiple SNPs in combination for Mendelian randomization has been used for causal inference for a broad range of environmental exposures and diseases (including BMI^39^ and lipids^17^) and use of an allele score in this regard yields reliable causal estimates.^40^

One limitation of our study was that we were unable to explore additional biological mechanisms, which might also explain the causal effects of height on CHD. Second, we rescaled the IV effect for comparability with the published ERFC results, which is only valid when assuming a linear association between the height and the IV effect. Third, the absence of association of our genetic instrument with risk of stroke should be interpreted with caution: additional Mendelian randomization studies using multiple instruments in larger sample sizes (such as METASTROKE) are needed to clarify the effect of adult height on stroke. Interesting next steps would include expanding this approach to cancer, as well as exploring the potential effect modification that adverse conditions during pregnancy or in early childhood may have on the associations of health outcomes with genetic instruments for completed growth.^41^

### Summary

Our multiple instruments Mendelian randomization approach provided evidence that people with a genetic predisposition to achieve a higher completed growth, measured by adult height, have a reduced risk of CHD, and with potential mechanisms including better lung function and lower levels of body mass index, non-HDL cholesterol, triglycerides and blood pressure.

## Funding and Acknowledgments

E.N. was a recipient of a Marie Curie Intra-European Fellowship for Career Development (grant No FP7-PEOPLE-2010-IEF-273673). F.W.A.is supported by UCL Hospitals NIHR Biomedical Research Centre. G.K.H. is recipient of a VENI grant (NWO nr91612122). S.S. received a grant from Ipse Movet. T.G., D.A.L. and G.D.S. work in a unit that is supported by the Medical Research Council (MC_UU_12013/1, 5 and 8). M.V.H. was supported by a Medical Research Council Population Health Scientist Fellowship (G0802432).

BRHS (British Regional Heart Study). BRHS has been funded principally by a series of programme and project grants from the British Heart Foundation (BHF), with additional support from the UK Medical Research Council, the Department of Health (England), the Institute of Alcohol Studies, the Stroke Association, the BUPA Foundation, the Wellcome Trust and the National Institute for Health Research School of Primary Care Research. DNA extraction was funded by a BHF Senior Fellowship.

BWHHS (British Women's Heart and Health Study). BWHHS is supported by funding from the British Heart Foundation and the Department of Health Policy Research Programme (England). We thank the BWHHS data collection team, general practitioners who helped with recruitment of participants and the participants. We thank all of the participants and the general practitioners, research nurses and data management staff who supported data collection and preparation. The BWHHS is coordinated by Shah Ebrahim (PI), D.L., and J.-P.C., with genotyping funded by the BHF (PG/07/131/24254, PI T.G.).

CAPS (The Caerphilly Prospective study). The CAPS study was undertaken by the former MRC Epidemiology Unit (South Wales) and was funded by the Medical Research Council of the UK. The DNA bank was established with funding from a MRC project grant. The data archive is maintained by the University of Bristol.

The IBC array data (also known as ‘Cardiochip' or ‘CVDSNP55v1_A' from the National Heart, Lung and Blood Institute (NHLBI) Candidate Gene Association Resource (CARe) was downloaded with appropriate permissions from The database of Genotypes and Phenotypes (dbGaP) [www.ncbi.nlm.gov/gap].

CARe (Candidate gene Association Resource). The CARe Consortium wishes to acknowledge the support of the National Heart, Lung, and Blood Institute and the contributions of the research institutions, study investigators, field staff and study participants in creating this resource for biomedical research (NHLBI contract number HHSN268200960009C). The following nine parent studies have contributed parent study data, ancillary study data and DNA samples through the Massachusetts Institute of Technology-Broad Institute (N01-HC-65226) to create this genotype/phenotype database for wide dissemination to the biomedical research community: the Atherosclerosis Risk in Communities (ARIC) study, the Cardiovascular Health Study (CHS), the Cleveland Family Study (CFS), the Cooperative Study of Sickle Cell Disease (CSSCD), the Coronary Artery Risk Development in Young Adults (CARDIA) study, the Framingham Heart Study (FHS), the Jackson Heart Study (JHS), the Multi-Ethnic Study of Atherosclerosis (MESA) and the Sleep Heart Health Study (SHHS). The ARIC study is carried out as a collaborative study supported by National Heart, Lung, and Blood Institute contracts N01-HC-55015, N01-HC-55016, N01-HC-55018, N01-HC-55019, N01-HC-55020, N01-HC-55021, and N01-HC-55022. The authors thank the staff and participants of the ARIC study for their important contributions. MESA was conducted and supported by contracts N01-HC-95159 through N01-HC-95169 and RR-024156 from the National Heart, Lung, and Blood Institute (NHLBI). The authors thank the participants of the MESA study, the Coordinating Center, MESA investigators and study staff for their valuable contributions. A full list of participating MESA investigators and institutions can be found at: [http://www.mesa-nhlbi.org].

EAS (Edinburgh Artery Study). EAS is funded by the British Heart Foundation (Programme Grant RG/98002), with Metabochip genotyping funded by a project grant from the Chief Scientist Office of Scotland (Project Grant CZB/4/672).

ELSA (English Longitudinal Study of Ageing). ELSA is funded by the National Institute on Aging in the US (R01 AG017644; R01AG1764406S1) and by a consortium of UK Government departments involved in areas related to the ageing process (including: Department for Communities and Local Government, Department for Transport, Department for Work and Pensions, Department of Health, HM Revenue and Customs and Office for National Statistics). ELSA was developed by a team of researchers based at the National Centre for Social Research, University College London and the Institute of Fiscal Studies. The data were collected by the National Centre for Social Research.

EPIC-NL (European Prospective Investigation into Cancer and Nutrition in The Netherlands). The EPIC-NL study was funded by ‘Europe against Cancer' Programme of the European Commission (SANCO), Dutch Ministry of Public Health, Welfare and Sports (VWS), Netherlands Cancer Registry (NKR), LK Research Funds, Dutch Prevention Funds, Dutch Cancer Society, ZonMW, The Netherlands Organisation for Health Research and Development and World Cancer Research Fund (WCRF) (The Netherlands). Genotyping was funded by IOP Genomics grant IGE05012 from Agentschap NL.

ET2DS is funded by the Medical Research Council (Project Grant G0500877), the Chief Scientist Office of Scottish (Programme Support Grant CZQ/1/38), Pfizer plc (Unrestricted Investigator Led Grant) and Diabetes UK (Clinical Research Fellowship 10/0003985). Research clinics were held at the Wellcome Trust Clinical Research Facility and Princess Alexandra Eye Pavilion in Edinburgh.

LURIC was supported by the 7th Framework Program (integrated project AtheroRemo, grant agreement number 201668 and RiskyCAD, grant agreement number 305739) of the European Union and by the INTERREG IV Oberrhein Program (Project A28, Genetic mechanisms of cardiovascular diseases) with support from the European Regional Development Fund (ERDF) and the Wissenschaftsoffensive TMO. NORDIL (Nordic Diltiazem study). The NORDIL clinical study was supported by a grant from Pharmacia. Genetic studies were supported by the British Heart Foundation (grant number CH/98001, RG/07/005/23633 to A.F.D.) and European Union Ingenious HyperCare Consortium: Integrated Genomics, Clinical Research, and Care in Hypertension (grant number LSHM-C7-2006-037093). Genotyping was supported by the British Heart Foundation (grant number PG/07/131/24254 to P.B.M.). We thank Prof. Thomas Hedner (Department of Clinical Pharmacology, Sahlgrenska Academy, Gotheburg, Sweden) and Prof. Sverre Kjeldsen (Ullevaal University Hospital, University of Oslo, Oslo, Norway), who are investigators of the NORDIL study.

PROCARDIS (Precocious Coronary Artery Disease). The PROCARDIS consortium genotyping was funded by the British Heart Foundation (BHF) and EC Sixth Framework Programme (LSHM-CT-2007-037273) and the sample collection by AstraZeneca AB and the BHF. R.C., M.F. and H.W. are supported by the BHF Centre for Research Excellence; M.F. and H.W. acknowledge support from a Wellcome Trust core award (090532/Z/09/Z). R.C. acknowledges support from the MRC; Anders Hamsten obtained support for this project from the Swedish Heart-Lung Foundation, the Swedish Medical Research Council (8691), the Knut and Alice Wallenberg Foundation, the Karolinska Institute and the Stockholm County Council (560183).

UCLEB (University College London-London School of Hygiene and Tropical Medicine-Edinburgh-Bristol). The UCLEB consortium is funded by a British Heart Foundation programme grant (ref RG/10/12/28456).

WHI (Women's Health Initiative). The WHI programme is funded by the National Heart, Lung, and Blood Institute, National Institutes of Health, U.S. Department of Health and Human Services through contracts N01WH22110, 24152, 32100-2, 32105-6, 32108-9, 32111-13, 32115, 32118-32119, 32122, 42107-26, 42129-32, and 44221.

WHII (Whitehall II study). The WHII study is supported by grants from the Medical Research Council (G0902037; ID85374), British Heart Foundation (RG/07/008/23674), Stroke Association, National Heart Lung and Blood Institute (5RO1 HL036310), National Institute on Aging (5RO1AG13196) Agency for Health Care Policy Research (HS06516), and the John D. and Catherine T. MacArthur Foundation Research Networks on Successful Midlife Development and Socio-economic Status and Health. We gratefully thank the subjects and the investigators of this project.

The funders had no role in study design, data collection and analysis, decision to publish or preparation of the manuscript.

**Conflict of interest:** Anke H Maitland-van der Zee has an unrestricted research grant for research on pharmacogenetics from GSK. All other primary authors declare no conflicts of interest.

Key Messages

- Observational studies show associations of adult height with risk of coronary heart disease (CHD) and stroke; however, these associations could arise from confounding or reverse causality
- To investigate the causal effect, we conducted a multi-locus Mendelian randomization study incorporating data from 180 height-related SNPs using both individual participant data from prospective cohorts and summary data from large genetics consortia
- A 6.5-cm increase in adult height (instrumented by 180 SNPs) causally reduced the odds of CHD by 10%, with potential mechanisms including blood pressure, body mass index and non-HDL cholesterol; the effect of adult height on stroke was less clear.

## Supplementary Material

Supplementary Data
