# Supplementary material for: Adult height, coronary heart disease and stroke: a multi-locus Mendelian randomization meta-analysis
Source: Int J Epidemiol. 2015 May 15;45(6):1927–37. doi: 10.1093/ije/dyv074 (PMC5841831; doi:10.1093/ije/dyv074)
Supplement: Supplementary Data [file dyv074_supplementary_data.zip › supplementary_data_April15th_CLEAN.docx]

**TABLE S1:** Details of included studies

AMS-PAS

Patients who presented at the Atherosclerosis Outpatient Clinic of the Academic Medical Center in Amsterdam, the Netherlands from 1995 to 2005 with a premature vascular event were recruited for participation in the cross-sectional AMC-PAS study. Patients qualified for inclusion once they had suffered from a documented myocardial infarction, surgical or percutaneous coronary revascularization, a coronary angiogram with evidence of at least a 70% stenosis in a major epicardial artery or stroke before the age of 51 years. From this study, comprising 1089 patients, a total of 743 samples passed QC. The Institutional Review Board approved the protocol (MEC04/236). All patients gave informed consent.

ARIC

The Atherosclerosis Risk In Communities (ARIC) Study is a population-based prospective cohort study of cardiovascular disease sponsored by the National Heart, Lung, and Blood Institute (NHLBI). ARIC originally included 15,792 individuals aged 45-64 years at baseline (1987-89), chosen by probability sampling from four US communities. Cohort members completed four clinic examinations each spread over about three years, conducted approximately three years apart between 1987 and 1998. The data used in this study are from the first visit in 1987-1989. A detailed study protocol is available on the ARIC study website (http://www.cscc.unc.edu/aric). For this study the sample was restricted to individuals of European descent by self-report and principal component analysis using genome-wide genotypes.

BRHS

From 1978 to 1980, 7735 men aged 40-59 were recruited from general practices across the UK for the British Regional Heart Study (BRHS). A wide range of phenotypic measures is available for established risk markers such as lipids, blood pressure and inflammatory and haemostatic markers. Most of these measures were taken both at recruitment and re-examination, which occurred in 1998-2000 when men were aged 60-79. At this re-examination 4252 participants attended and DNA was extracted for 3945. Data on important behavioural variables such as cigarette and alcohol consumption, as well as physical activity, have been regularly collected through follow up. Well validated outcome variables including major coronary heart disease and stroke, as well as cause-specific mortality, continue to be collected from medical records 30 years after recruitment.

BWHHS

The British Women’s Heart & Health Study (BWHHS) is a prospective cohort study of 4,286 women aged between 60 and 79 at baseline in 1999-2000. Participants were randomly selected from general practice registers in 23 towns across England, Wales and Scotland. The criteria for selection the town, GP practise and participants were based on the BRHS. Baseline measurements were taken in 1999/2000 with follow-up questionnaires in 2003, 2007 and 2010-2011. Biomarkers and blood samples for DNA were measured by research nurses at baseline interview. Survival status is obtained from the Data Linkage Service, Health and Social Care Information Centre, London and CVD events have been prospectively studied by GP record review. Of the 4,278 participants who gave consent for genetic studies, 15 were defined by the examining nurse as being non-white and were excluded from further analysis. Of the remaining 4,263 women, 3,800 (89%) had DNA available for genotyping.

CAPS

The Caerphilly Prospective Study (CAPS) to examine the importance of lipids, haemostatic factors, and hormones such as testosterone, cortisol and insulin (Lichtenstein et al 1987) in the development of ischemic heart disease (IHD). The initial design attempted to contact all men aged 45 to 59 years from the town of Caerphilly and adjoining villages. 2512 subjects (response rate 89%) identified from the electoral register and general practice lists were examined between July 1979 and September 1983 (phase I). Men were initially seen at an evening clinic, where they completed a questionnaire, had anthropometric measures and an ECG taken. They also completed a food frequency questionnaire at home. They subsequently re-attended an early morning clinic to have fasting blood samples for a wide variety of tests. Quality control was examined by the use of both "blind" split samples as well as a second repeat measure on a random sub-sample to examine intra-individual variation.

CARDIA

The Coronary Artery Risk Development in Young Adults (CARDIA) Study is a study examining the development and determinants of clinical and subclinical cardiovascular disease and its risk factors. It began in 1985 with a group of 5115 black and white men and women aged 18-30 years. The participants were selected so that there would be approximately the same number of people in subgroups of race, gender, education (high school or less and more than high school) and age (18-24 and 25-30) in each of 4 centers: Birmingham, AL; Chicago, IL; Minneapolis, MN; and Oakland, CA. These same participants were asked to participate in follow-up examinations during 1987-1988 (Year 2), 1990-1991 (Year 5), 1992-1993 (Year 7), 1995-1996 (Year 10), 2000-2001 (Year 15), 2005-2006 (Year 20), and 2010-2011 (Year 25). A majority of the group has been examined at each of the follow-up examinations (90%, 86%, 81%, 79%, 74%, 72%, and 70%, respectively). The sample was restricted to individuals of European descent by self-report and principal component analysis using genome-wide genotypes.

CHS

The Cardiovascular Health Study (CHS) is a population-based cohort study of risk factors for cardiovascular disease in adults 65 years of age or older conducted across four field centres. The original predominantly white cohort of 5,201 persons was recruited in 1989-1990 from random samples of the Medicare eligibility lists and an additional 687 African-Americans were enrolled in 1992-93 for a total sample of 5,888. The sample was restricted to individuals of European descent by self-report and principal component analysis using genome-wide genotypes.

EAS

The Edinburgh Artery Study (EAS) is an age-stratified random sample of men and women, aged 55-74 years, which was selected between August 1987 and September 1988 from the age-sex registers of ten general practices with a geographical and socio-economical catchment population spread throughout the city of Edinburgh, UK. Subjects were excluded if they were unfit to participate (e.g. due to severe mental illness or terminal disease); excluded individuals were replaced by other randomly sampled subjects.

ELSA

The English Longitudinal Study of Ageing (ELSA) is a national cohort of participants (48% men) aged over 50 years recruited from the Health Surveys for England in 1998, 1999, and 2001. Genetic data were collected at wave 2 of the study (2004/5); the phenotype measurements taken at wave 2 were used for this study.

EPIC-NL

The European Prospective Investigation of Cancer (EPIC) study in The Netherlands is based in two centres, Bilthoven and Utrecht. The population in the two cohorts has been recruited from two regions, from the general population (Bilthoven) and from those attending for breast cancer screening (Utrecht). Recruitment was carried out between 1993 and 1997. In 2006-2007, the two Dutch cohorts have been merged into one cohort (www.epicnl.eu) to gain efficiency and sample size and to optimise the use of the data locally. The separate cohorts, however, will co-exist besides the merged cohort.

ET2DS

The Edinburgh Type 2 Diabetes Study (ET2DS) is based on an age-stratified random sample of men and women with type 2 diabetes, aged 60–74 years, which was selected between August 2006 and August 2007 from the Lothian Diabetes Register (LDR), a comprehensive database of subjects with known type 2 diabetes living in Lothian. Subjects were excluded if they did not meet WHO criteria for type 2 diabetes, or if they were physically unable to complete the clinical and cognitive examination. The study population is almost exclusively European. DNA was extracted at baseline. Physical examinations were performed by specially trained research nurses using standardised operating procedures. The quality of measurements was checked using observation of research staff by study investigators and inter-observer variability assessments were made for key variables. Blood assays were performed in accredited laboratories using international standards. Retrospective data on cardiovascular disease and selected physical and biochemical variables were retrieved using record linkage for hospitalisations and deaths since 1985 and using data from the LDR. Subjects returned for further clinical examination after one year and were examined again after they had participated for 4 years.

FHS

The Framingham Heart Study (FHS) began in 1948 with the recruitment of an original cohort of 5,209 men and women (mean age 44 years; 55% women). In 1971 a second generation of study participants was enrolled; this cohort consisted of 5,124 children and spouses of children of the original cohort. The mean age of the offspring cohort was 37 years; 52% were women. A third generation cohort of 4,095 children of offspring cohort participants (mean age 40 years; 53% women) was enrolled beginning in 2002. Details of study designs for the three cohorts are summarized elsewhere. At each clinic visit, a medical history was obtained with a focus on cardiovascular content. The sample was restricted to individuals of European descent by self-report and principal component analysis using genome-wide genotypes. In the case of related individuals, only the oldest individual in each family unit was included in the analysis.

GIRaFH

The Genetic Identification of Risk Factors in Familial Hypercholesterolemia (GIRaFH) is a retrospective multicenter cohort study, described in detail by Jansen AC et al (J Intern Med 2004;256(6):482-90). The diagnosis of FH was based upon the presence of a low-density lipoprotein receptor mutation or upon strict clinical criteria Dutch FH criteria). Clinical events were defined according to the criteria used in the AMC PAS cohort, and the data with respect to CVD events were collected by scrutinizing medical records and the use of questionnaires. A total of 2400 FH patients from 27 Dutch lipid clinics were included in the initial cohort. A total of 1489 of the samples available for genotyping passed QC. The local Institutional Review Board approved the protocol. All patients gave informed consent.

LURIC

The Ludwigshafen Risk and Cardiovascular Health (LURIC) study is an ongoing prospective study of 3316 patients recruited at the cardiac centre in Ludwigshafen (Germany) between 1997 and 2000. The cardiovascular and metabolic phenotypes (CAD, MI, dyslipidaemia, hypertension, metabolic syndrome and diabetes mellitus) have been defined or ruled out using standardised methodologies in all study participants. Inclusion criteria for LURIC were German ancestry, clinical stability and availability of a coronary angiogram. Exclusion criteria were any acute illness other than ACS, any chronic disease where non-cardiac disease predominated or a history of malignancy within the last five years. After written informed consent baseline examination was done consisting of a standardised individual and family history questionnaire and extensive sampling of fasted venous blood in the early morning. To all individuals without known diabetes mellitus and insulin therapy an oral glucose tolerance test was offered.

MDC

The Malmo Diet and Cancer (MDC) study is set in Malmö, Sweden's third largest city. The background population consisted of all men born between 1923 and 1945 and all women born between 1923 and 1950 who were living in Malmö during the screening period 1991 to 1996 (n = 74,138). This population was identified through the Swedish national population registries. The final cohort consisted of 28,098 individuals (participation rate 40.8%). The subjects were recruited through advertisements in local media and through invitation by mail. The only exclusion criteria were inadequate Swedish language skills and mental incapacity. The Ethics Committee at Lund University approved the design of the MDC study (LU 51–90). Written informed consent was obtained from the participants.

MESA

The Multi-Ethnic Study of Atherosclerosis (MESA) investigation is a population-based study of 6,814 men and women age 45 to 85 years, without clinical cardiovascular disease, recruited from six United States communities (Baltimore, MD; Chicago, IL; Forsyth County, NC; Los Angeles County, CA; northern Manhattan, NY; and St. Paul, MN). The main objective of MESA is to determine the characteristics of subclinical cardiovascular disease and its progression. Sampling and recruitment procedures have been previously described in detail57. Adults with symptoms or history of medical or surgical treatment for cardiovascular disease were excluded. During the recruitment process, potential participants were asked about their race/ethnicity. Self-reported ethnicity was used to classify participants into groups. Additional individuals were derived from the MESA Family Study, an ancillary study to MESA whose goal is to identify genes contributing to the risk for cardiovascular disease, by looking at the early manifestations of atherosclerosis within families, mainly siblings. MESA Family studied siblings of index subjects from the MESA study and sib-pairs in new families ascertained through index subjects meeting MESA enrolment criteria. In a small proportion of subjects, parents of MESA index subjects participating in MESA Family were studied but only to have blood drawn for genotyping. The MESA Family cohort was recruited from the six MESA Field Centers during May 2004 - May 2007. The number of non-classic MESA family members recruited was 1,633 (950 African-Americans and 683 Hispanic-Americans) from 594 families, yielding 3,026 sib-pairs. Participants underwent the same examination as MESA participants. The sample was restricted to individuals of European descent by self-report and principal component analysis using genome-wide genotypes.

NORDIL

The Nordic Diltiazem intervention study (NORDIL) was started in September 1992. This trial was a prospective randomized open blinded-endpoint multicenter, parallel-group study conducted in Norway and Sweden. The study was designed to evaluate the potential preventive effects of diltiazem compared with conventional antihypertensive drug treatment. Primary endpoints were cardiovascular mortality defined as fatal acute myocardial infarction, fatal acute cerebrovascular disease (stroke), sudden death and other fatal cardiovascular disease as well as cardiovascular morbidity defined as myocardial infarction and cerebrovascular disease (stroke). Secondary endpoints are total mortality, the development or deterioration of ischemic heart disease, congestive heart failure, atrial fibrillation, transient ischemic attacks, diabetes mellitus and renal insufficiency. Male and female patients, aged 50-69, with primary hypertension were randomly allocated to therapy starting with either diltiazem (180-360 mg daily) or conventional treatment (diuretics or beta-adrenergic blockers). Add-on therapy in the conventional treatment group excluded all types of calcium antagonists. The goal of treatment was a target diastolic blood pressure of ≤90 mmHg or a 10% diastolic blood pressure reduction.

PROCARDIS

The Precocious Coronary Artery Disease study (PROCARDIS) is a European consortium investigating the genetics of coronary artery disease (CAD) in German, Italian, Swedish, and British CAD patients and controls. Controls in this study had no personal history of CAD, hypertension, or diabetes. Ascertainment criteria for PROCARDIS probands were MI or symptomatic ACS (SACS), on the assumption that the latter represents a similar pathological process according to modified World Health Organisation diagnostic criteria before the age of 66 y. Diagnosis of MI required documentation of two or more of: (a) typical ischemic chest pain, pulmonary oedema, syncope or shock; (b) development of pathological Q-waves and/or appearance or disappearance of localized ST-elevation followed by T-wave inversion in two or more standard electrocardiograph leads; (c) increase in concentration of serum enzymes consistent with MI (e.g. creatine kinase more than twice the upper limit of normal). Diagnosis of SACS required documentation of hospitalization for one of the following indications: (a) unstable angina diagnosed by typical ischemic chest pain at rest associated with reversible ST-depression in two or more standard electrocardiograph leads; (b) thrombolysis for suspected MI (as indicated by localized ST-elevation in two or more standard electrocardiograph leads) even without later development of T-wave inversion, Q-waves, or a significant enzyme rise; or (c) emergency revascularization (i.e. during same admission) following presentation with typical ischemic chest pain at rest. Probands completed questionnaires in order to recruit affected siblings with a range of CAD diagnoses at age <66 y (MI, SACS, chronic stable angina, or intervention for coronary revascularization), who were then invited to participate in the study if their diagnoses were confirmed. Parents and up to four unaffected siblings per family were recruited wherever possible to augment the recovery of linkage phase information. Informative families were recruited in Germany, Italy, Sweden, and the United Kingdom; 99.5% of the study participants reported having a white European ancestry. The protocol was approved by the Ethics Committees of the participating institutions and all participants gave written, informed consent.

UCP

The Utrecht Cardiovascular PHarmacogenetics (UCP) study enrolled participants from the population-based Pharmaco-Morbidity Record Linkage System (PHARMO, www.pharmo.nl). PHARMO links drug dispensing histories from a representative sample of Dutch community pharmacies to the national registration of hospital discharges (Dutch National Medical Registry). First, patients who received a prescription for an antihypertensive drug, and/or had hypercholesterolemia (prescription for a cholesterol-lowering drug or total cholesterol >5.0mmol/l), were selected from the PHARMO database for pharmacogenetic studies on antihypertensive drugs and statins, respectively. From this cohort, a nested case–control study was designed using hospital discharge records. Patients hospitalized for MI [International Classification of Diseases 9 code 410] were included as cases if they were registered in PHARMO for at least 1 year and were older than 18 years. The index date was defined as the date of hospitalization for the first MI. Controls met the same eligibility criteria as the cases, but had not developed MI. Controls were matched with cases on age, sex, and region, and assigned the same index date. The sample was restricted to individuals of European descent by principal component analysis using genome-wide genotypes.

WHI

WHI is one of the largest (n = 161,808) studies of women’s health ever undertaken in the U.S. There are two major components of WHI: (1) a clinical trial (CT) that enrolled and randomized 68,132 women ages 50–79 into at least one of three placebo-control clinical trials (hormone therapy, dietary modification, and supplementation with calcium and vitamin D); and (2) an observational study (OS) that enrolled 93,676 women of the same age range into a parallel prospective cohort study.

WH-II

The Whitehall II (WH-II) Study recruited 10,308 participants (70% men) between 1985 and 1989 from 20 London based civil service departments. In this longitudinal study blood pressure was recorded at phase 1 (1985-1988), phase 3 (1991-1993), phase 5 (1997-1999) and phase 7 (2003-2004). DNA was stored from phase 7 from over 6,000 participants. The study participants are all highly phenotyped for cardiovascular and other ageing related health outcomes.

**Table S2.** Characteristics of 21 included studies

| **Study** | **Country** | **Study design** | **Sampling frame** | **Genotyping platform** | **Baseline  year(s)** | **Sample size** | **CHD  (cases [incident])** | **Stroke (cases [incident])** | **Study Reference (PMID)** |
| --- | --- | --- | --- | --- | --- | --- | --- | --- | --- |
|  |  |  |  |  |  |  |  |  |  |
| AMC-PAS | NL | Cases only | Clinic | IBC Cardiochip | 1993 | 743 | 552 (0) | 27 (0) | 19164808 |
| ARIC | USA | Cohort | Community | IBC Cardiochip | 1986 | 9206 | 985 (726) | 510 (376) | 20400780 |
| BRHS | UK | Cohort | General practices | Metabochip | 1998-2000 | 2329 | 344 (184) | 112 (112) | 6789956 |
| BWHHS | UK | Cohort | Population | IBC Cardiochip | 1999-2001 | 3385 | 138 (50) | 114 (66) | 16045529 |
| CAPS | UK | Cohort | General practices | Metabochip | 1993-1994 | 1349 | NA | 200 (60) | 6332166 |
| CARDIA | USA | Cohort | Community | IBC Cardiochip | 1984 | 1292 | 10 (0) | 4 (0) | 20400780 |
| CHS | USA | Cohort | Community | IBC Cardiochip | 1989 | 3495 | 903 (523) | 727 (585) | 20400780 |
| EAS | UK | Cohort | General practices | Metabochip | 2004 | 764 | NA | 100 (93) | 1917239 |
| ELSA | UK | Cohort | Respondents of HSE | Metabochip | 2004 | 1883 | 230 (54) | 76 (76) | 23143611 |
| EPIC-NL | NL | Nested case-control | Population | IBC Cardiochip | 1993-1997 | 5192 | 1222 (1222) | 443 (443) | 19483199 |
| ET2DS | UK | Cohort | Diabetes register | Metabochip | 2006-2007 | 1007 | 154 (149) | NA | 19077235 |
| FHS | USA | Cohort | Community | IBC Cardiochip | 1948/1971/2002 | 657 | 28 (0) | 37 (0) | 20400780 |
| GIRAFH | NL | Cohort | Clinic | IBC Cardiochip | 1999 | 1456 | 239 (239) | 27 (27) | 15554949 |
| LURIC | GER | Case-control | Population | IBC Cardiochip | 1997 | 2778 | 1378 (476) | 268 (52) | 19164808 |
| MDC | UK/SWE | RCT | Population | IBC Cardiochip | 1991 | 1937 | 57 (57) | 46 (46) | 8429286 |
| MESA | USA | Cohort | Community | IBC Cardiochip | 2000 | 2217 | 47 (0) | 32 (0) | 20400780 |
| NORDIL | UK/SWE | RCT | Population | IBC Cardiochip | 1992 | 969 | 23 (23) | 26 (26) | 8173702 |
| PROCARDIS | UK | Case-control | Clinic | IBC Cardiochip | 1998 | 4944 | 1498 (0) | NA | 15266304 |
| UCP | NL | Nested case-control | Clinic | IBC Cardiochip | 2007 | 1589 | 632 (632) | NA | 20712525 |
| WHI | USA | Nested case-control | Community | IBC Cardiochip | 1994-1998 | 7878 | 2376 (2376) | 2129 (2129) | 9492970 |
| WH-II | UK | Cohort | Workplace | IBC Cardiochip | 1985-1988 | 4958 | 32 (0) | NA | 1674771 |
|  |  |  |  |  |  |  |  |  |  |

AMC-PAS, Academic Medical Center Amsterdam Premature Artherosclerosis Study. ARIC, Atherosclerosis Risk In Communities. BWHHS, British Women's Heart and Health Study. CARDIA, Coronary Artery Risk Development in Young Adults. CHS, Cardiovascular Health Study. EPIC-NL, European Prospective Investigation into Cancer and Nutrition in the Netherlands. FHS, Framingham Heart Study. GIRAFH, Genetic Identification of Riskfactors in Familial Hypercholesterolemia. LURIC, Ludwigshafen Risk and Cardiovascular Health Study. MDC, Malmo Diet and Cancer (MDC) Study. MESA, Multi-Ethnic Study of Atherosclerosis. NORDIL, Nordic Diltiazem (NORDIL) Study. PROCARDIS, Precocious Coronary Artery Disease. SMART, Second Manifestations of ARTerial disease. UCP, Utrecht Cardiovascular Pharmacogenetics. WHI, Women's Health Initiative. WH-II, Whitehall II study. PMID, Pubmed identifier.

**TABLE S3.** Definitions of cardiovascular disease events in individual studies

|  | **CHD** | | | | | **Stroke** | | | | |
| --- | --- | --- | --- | --- | --- | --- | --- | --- | --- | --- |
|  | **Non-fatal** | | | **Fatal** | | **Non-fatal** | | | **Fatal** | |
| **Study** | **Self report** | **Medical records** | **Clinical/lab. measures** | **Death certificate** | **ICD coded** | **Self report** | **Medical records** | **Clinical/lab. /imaging measures** | **Death certificate** | **ICD coded** |
| **AMC-PAS** |  | ● | ● |  |  |  | ● | ● |  |  |
| **ARIC** |  | ● |  | ● | ● |  | ● |  | ● | ● |
| **BRHS** | ● | ● |  | ● | ● | ● | ● |  | ● | ● |
| **BWHHS** | ● | ● |  | ● | ● | ● | ● |  | ● | ● |
| **CARDIA** |  | ● |  | ● |  |  | ● |  | ● |  |
| **CAPS** |  |  |  |  |  | ● | ● | ● | ● | ● |
| **CHS** | ● | ● |  | ● | ● | ● | ● |  | ● | ● |
| **EAS** |  | ● |  | ● | ● |  | ● |  | ● | ● |
| **ELSA** | ● |  |  | ● |  | ● |  |  | ● |  |
| **EPIC Netherlands** |  | ● |  | ● | ● |  | ● |  | ● | ● |
| **ET2DS** | ● | ● |  | ● | ● | ● | ● |  | ● | ● |
| **FHS** |  | ● |  | ● |  |  | ● |  | ● |  |
| **GIRAFH** |  | ● | ● |  |  |  | ● | ● |  |  |
| **LURIC** |  |  | ● | ● |  | ● |  |  | ● |  |
| **MDC** | ● | ● |  |  |  | ● | ● |  |  |  |
| **MESA** | ● |  |  |  |  | ● |  |  |  |  |
| **NORDIL** | ● | ● |  |  |  | ● | ● |  |  |  |
| **PROCARDIS** | ● | ● |  |  |  |  |  |  |  |  |
| **UCP** |  | ● |  |  |  |  |  |  |  |  |
| **WHI** | ● |  |  |  |  | ● |  |  |  |  |
| **WH II** |  | ● |  |  |  |  | ● |  |  |  |

**TABLE S4.** Cardiovascular risk factors in the 21 included studies

| **Study** | **Age, mean(SD)** | **Females,  %** | **Height, mean(SD)** | **BMI, mean(SD)** | **Smokers,  %** | **Systolic BP, mean(SD)** | **HDL cholesterol, mean(SD)** | **Non-HDL cholesterol, mean(SD)** | **Glucose, mean(SD)** | **Triglycerides, geometric mean(CV)** | **CRP,  geometric mean(CV)** |
| --- | --- | --- | --- | --- | --- | --- | --- | --- | --- | --- | --- |
|  |  |  |  |  |  |  |  |  |  |  |  |
| AMC_PAS | 43.0 (5.3) | 24 | 175.4 (9.3) | 26.9 (4.0) | 75 | 128.6 (18.1) | 1.15 (0.33) | 2.97 (1.22) | 5.69 (1.68) | 1.51 (1.93) | 3.70 (3.31) |
| ARIC | 54.3 (5.7) | 54 | 168.7 (9.4) | 27.0 (4.8) | 60 | 118.4 (17.0) | 1.31 (0.43) | 4.24 (1.12) | 5.91 (1.69) | 1.34 (1.68) | 2.25 (2.96) |
| BRHS | 68.9 (5.6) | 0 | 172.0 (6.6) | 26.8 (3.6) | 72 | 144.2 (20.0) | 1.15 (0.25) | 5.23 (1.06) | 5.56 (1.31) | 1.77 (1.71) | 0.80 (2.79) |
| BWHHS | 68.9 (5.5) | 100 | 158.8 (6.1) | 27.6 (4.9) | 44 | 147.0 (25.1) | 1.66 (0.45) | 2.46 (0.42) | 6.06 (1.64) | 1.66 (1.59) | 1.78 (3.16) |
| CAPS | 55.4 (9.6) | NA | 165.1 (32.7) | 25.6 (6.2) | 78 | 142.0 (32.7) | 0.97 (0.33) | 4.34 (1.43) | 5.07 (1.68) | 1.69 (1.66) | 0.73 (2.68) |
| CARDIA | 25.6 (3.4) | 54 | 171.3 (9.2) | 23.6 (4.0) | 24 | 109.3 (10.8) | 1.34 (0.33) | 3.22 (0.86) | 4.74 (0.97) | 0.76 (1.66) | 0.97 (3.30) |
| CHS | 72.8 (5.6) | 56 | 165.2 (9.4) | 26.4 (4.5) | 54 | 135.5 (21.5) | 1.38 (0.40) | 4.09 (1.00) | 6.04 (1.82) | 1.47 (1.54) | 1.88 (2.76) |
| EAS | 64.5 (5.6) | 48 | 164.3 (12.8) | 26.2 (4.5) | 62 | 147.7 (26.3) | 1.45 (0.39) | 5.63 (1.37) | 5.77 (1.39) | 1.38 (1.55) | 0.84 (2.79) |
| ELSA | 73.7 (9.4) | 53 | 155.6 (38.8) | 25.7 (8.1) | 67 | 122.7 (48.4) | 1.48 (0.40) | 4.19 (1.19) | 2.30 (2.60) | 1.57 (1.64) | 0.94 (2.30) |
| EPIC_NL | 54.1 (10.1) | 78 | 167.1 (8.3) | 26.8 (4.5) | 64 | 133.1 (21.2) | 1.40 (0.41) | 4.23 (1.25) | 5.41 (2.21) | 1.56 (1.75) | 2.30 (5.39) |
| ET2DS | 67.9 (4.2) | 51 | 165.9 (10.6) | 31.4 (5.7) | 61 | 133.2 (17.5) | 1.29 (0.38) | 3.00 (0.90) | 7.46 (2.25) | NA | 0.88 (2.68) |
| FHS | 45.7 (10.1) | 51 | 169.0 (9.0) | 27.7 (5.7) | 61 | 135.6 (20.4) | 1.32 (0.40) | 3.80 (1.09) | 5.60 (0.60) | 2.85 (1.95) | 1.51 (3.51) |
| GIRAFH | 44.6 (12.6) | 48 | 172.2 (9.4) | 25.1 (3.5) | 73 | 135.1 (19.7) | 1.22 (0.36) | 8.12 (1.93) | 5.10 (1.03) | 1.58 (1.65) | NA |
| LURIC | 62.8 (10.7) | 31 | 170.0 (8.8) | 27.4 (4.1) | 65 | 141.1 (23.5) | 1.01 (0.28) | 3.95 (0.97) | 6.30 (1.98) | 1.71 (1.62) | 3.56 (3.76) |
| MDC | 57.8 (5.8) | 57 | 169.5 (9.0) | 24.4 (3.3) | 67 | 115.7 (6.2) | NA | NA | NA | NA | NA |
| MESA | 62.7 (10.2) | 48 | 169.0 (9.6) | 27.7 (5.1) | 56 | 123.5 (20.7) | 1.35 (0.41) | 3.71 (0.93) | 5.04 (1.23) | 1.30 (1.71) | 1.78 (3.17) |
| NORDIL | 56.0 (4.0) | 51 | 170.6 (8.7) | 28.3 (4.6) | 26 | 177.2 (14.5) | 1.36 (0.46) | NA | 5.26 (1.54) | 1.60 (1.66) | NA |
| PROCARDIS | 60.7 (9.1) | 40 | 169.2 (9.4) | NA | NA | NA | NA | NA | NA | NA | NA |
| UCP | 62.8 (9.6) | 25 | 173.3 (8.5) | 27.1 (4.1) | 69 | NA | NA | NA | NA | NA | NA |
| WHI | 68.0 (6.6) | 100 | 161.0 (6.5) | 28.3 (6.2) | 50 | 132.9 (18.7) | 1.30 (0.31) | 4.05 (0.92) | 5.82 (1.90) | 1.55 (1.64) | 26.20 (2.88) |
| WHITEHALL_II | 60.9 (5.9) | 26 | 172.0 (8.9) | 26.7 (4.3) | 53 | 128.1 (16.6) | 1.58 (0.45) | 4.16 (1.04) | 5.44 (1.19) | 1.22 (1.67) | 1.31 (2.99) |
|  |  |  |  |  |  |  |  |  |  |  |  |

**TABLE S5.** Characteristics of 69 SNPs used for allele score construction

| **SNP** | **Chr location** | **Candidate gene** | **Imputation  R^2^** | **Effect allele** | **No studies** | **No participants** | **EAF** | **HWE*** | **Effect (95% CI)** | **P** | **Heterogeneity**** |
| --- | --- | --- | --- | --- | --- | --- | --- | --- | --- | --- | --- |
|  |  |  |  |  |  |  |  |  |  |  |  |
| rs10512248 | 9q22 | PTCH1 | 1.000 | C | 21 | 27311/27164/6952 | 0.555 | 0.56 | 0.22 (0.12, 0.33) | <0.001 | 13% (0.284) |
| rs1051431 | 12q24 | MPHOSPH9 | 0.946 | G | 20 | 36042/19823/2686 | 0.384 | 0.234 | 0.13 (0.01, 0.25) | 0.037 | 16% (0.259) |
| rs11024739 | 11p15 | SPTY2D1 | 0.998 | C | 21 | 33552/23597/4273 | 0.454 | 0.567 | 0.20 (0.10, 0.31) | <0.001 | 0% (0.880) |
| rs11096991 | 4p14 | RFC1 | N/A | G | 16 | 6461/24633/23016 | 0.881 | 0.56 | 0.08 (-0.03, 0.18) | 0.145 | 0% (0.980) |
| rs11144688 | 9q21 | PCSK5 | 1.000 | A | 21 | 878/13018/47573 | 0.986 | 0.469 | 0.30 (0.15, 0.44) | <0.001 | 0% (0.526) |
| rs1129923 | 1q23 | DUSP23 | N/A | A | 16 | 498/9171/44440 | 0.991 | 0.463 | 0.25 (0.08, 0.42) | 0.004 | 7% (0.371) |
| rs1150781 | 6p21 | HMGA1 | 0.930 | G | 19 | 43678/8819/4493 | 0.234 | 0.647 | 0.41 (0.24, 0.59) | <0.001 | 21% (0.203) |
| rs1173736 | 5p14 | NPR3 | 1.000 | G | 21 | 33308/23837/4300 | 0.458 | 0.335 | 0.20 (0.09, 0.31) | <0.001 | 0% (0.928) |
| rs12145922 | 1p22 | PKN2 | 0.976 | C | 21 | 11103/29291/19485 | 0.815 | 0.573 | 0.08 (-0.02, 0.18) | 0.108 | 3% (0.420) |
| rs12590407 | 14q11 | NFATC4 | 0.958 | G | 21 | 29357/24738/5228 | 0.505 | 0.321 | 0.19 (0.08, 0.29) | <0.001 | 0% (0.767) |
| rs1421811 | 5p14 | NPR3 | 1.000 | G | 21 | 22889/29098/9432 | 0.627 | 0.34 | 0.12 (0.02, 0.22) | 0.015 | 0% (0.722) |
| rs1476387 | 6q21 | ZBTB24 | 0.984 | A | 21 | 10626/29645/20791 | 0.826 | 0.36 | 0.19 (0.09, 0.29) | <0.001 | 0% (0.762) |
| rs1636255 | 7p22 | GNA12 | N/A | A | 16 | 4186/20412/28583 | 0.921 | 0.337 | 0.29 (0.17, 0.40) | <0.001 | 13% (0.309) |
| rs16942341 | 15q26 | ACAN | 0.997 | A | 21 | 52/3410/57999 | 0.999 | 0.737 | 0.98 (0.69, 1.26) | <0.001 | 18% (0.225) |
| rs17622208 | 5q31 | SLC22A5 | 0.980 | A | 21 | 17183/30401/13360 | 0.718 | 0.551 | 0.21 (0.11, 0.30) | <0.001 | 36% (0.051) |
| rs1800783 | 7q36 | ATG9B | 1.000 | T | 20 | 8427/27382/22124 | 0.855 | 0.61 | 0.21 (0.11, 0.31) | <0.001 | 3% (0.417) |
| rs1822469 | 2p15 | PPP3R1 | 0.920 | A | 20 | 8778/26978/20213 | 0.843 | 0.327 | 0.19 (0.09, 0.29) | <0.001 | 15% (0.270) |
| rs1866146 | 2p23 | POMC | 0.963 | G | 21 | 26972/26589/6737 | 0.553 | 0.391 | 0.25 (0.15, 0.35) | <0.001 | 12% (0.308) |
| rs2046159 | 1p22 | COL24A1 | 0.997 | G | 21 | 1677/16464/43275 | 0.973 | 0.484 | 0.11 (-0.02, 0.23) | 0.108 | 29% (0.106) |
| rs2075870 | 11p15 | KCNQ1 | 0.983 | A | 21 | 7046/2917/51469 | 0.885 | 0.511 | 0.74 (0.44, 1.05) | <0.001 | 39% (0.034) |
| rs2284746 | 1p36 | MFAP2 | 0.940 | C | 20 | 13146/28682/15541 | 0.771 | 0.491 | 0.26 (0.16, 0.36) | <0.001 | 24% (0.160) |
| rs2425012 | 20q11 | MYH7B | 0.993 | A | 21 | 11149/29642/20385 | 0.818 | 0.433 | 0.18 (0.08, 0.28) | <0.001 | 0% (0.867) |
| rs2425019 | 20q11 | MMP24 | 1.000 | G | 21 | 18240/30191/13005 | 0.703 | 0.335 | 0.29 (0.20, 0.39) | <0.001 | 0% (0.629) |
| rs2516448 | 6p21 | MICA | 1.000 | A | 16 | 12666/21832/10084 | 0.716 | 0.472 | 0.47 (0.37, 0.58) | <0.001 | 64% (<0.001) |
| rs258281 | 16p13 | CASKIN1 | 0.974 | A | 21 | 2004/17927/40947 | 0.967 | 0.56 | 0.21 (0.09, 0.33) | 0.001 | 0% (0.904) |
| rs2596494 | 6p21 | HLA-B | 0.867 | C | 19 | 30911/13601/1689 | 0.331 | 0.188 | 0.54 (0.41, 0.68) | <0.001 | 40% (0.038) |
| rs2679178 | 2q24 | NPPC | 0.955 | A | 21 | 424/9149/51499 | 0.993 | 0.568 | 0.49 (0.32, 0.66) | <0.001 | 23% (0.167) |
| rs2871865 | 15q26 | IGF1R | N/A | G | 16 | 730/10711/42660 | 0.987 | 0.353 | 0.51 (0.36, 0.67) | <0.001 | 0% (0.805) |
| rs291700 | 20q11 | CDK5RAP1 | 0.872 | A | 20 | 5317/23555/26173 | 0.903 | 0.342 | 0.24 (0.14, 0.35) | <0.001 | 51% (0.004) |
| rs3103296 | 2q37 | DIS3L2 | 0.916 | A | 20 | 7566/26229/22784 | 0.866 | 0.265 | 0.16 (0.06, 0.26) | 0.002 | 8% (0.351) |
| rs3107179 | 2q24 | NPPC | N/A | G | 16 | 8255/25550/20310 | 0.847 | 0.613 | 0.29 (0.19, 0.39) | <0.001 | 51% (0.010) |
| rs3210043 | 14q11 | BCL2L2 | N/A | A | 16 | 38575/14206/1335 | 0.287 | 0.542 | 0.27 (0.13, 0.41) | <0.001 | 0% (0.551) |
| rs35874463 | 15q22 | SMAD3 | N/A | G | 15 | 40293/4423/130 | 0.102 | 0.743 | 0.57 (0.33, 0.81) | <0.001 | 0% (0.533) |
| rs3734254 | 6p21 | PPARD | 0.975 | G | 20 | 2653/19195/37323 | 0.955 | 0.59 | 0.44 (0.32, 0.56) | <0.001 | 0% (0.710) |
| rs3751591 | 15q21 | CYP19A1 | N/A | G | 16 | 1595/15156/37366 | 0.971 | 0.503 | 0.24 (0.10, 0.37) | <0.001 | 35% (0.081) |
| rs3782415 | 12q22 | SOCS2 | 0.999 | G | 21 | 38665/20096/2669 | 0.371 | 0.479 | 0.43 (0.32, 0.55) | <0.001 | 0% (0.867) |
| rs3796529 | 14p11 | REST | N/A | A | 16 | 35070/16591/2041 | 0.347 | 0.468 | 0.23 (0.10, 0.35) | <0.001 | 0% (0.600) |
| rs3812265 | 7q22 | CNOT4 | 0.822 | A | 20 | 31057/21121/3291 | 0.44 | 0.139 | 0.25 (0.13, 0.36) | <0.001 | 0% (0.751) |
| rs3816540 | 1p36 | RPS6KA1 | N/A | C | 15 | 2684/18084/31928 | 0.949 | 0.41 | 0.23 (0.11, 0.35) | <0.001 | 28% (0.152) |
| rs4252548 | 19q13 | IL11 | N/A | A | 16 | 40/2464/51588 | 0.999 | 0.571 | 0.59 (0.26, 0.91) | <0.001 | 0% (0.978) |
| rs4272 | 7q22 | CDK6 | 1.000 | G | 21 | 37901/20756/2774 | 0.383 | 0.498 | 0.41 (0.30, 0.53) | <0.001 | 0% (0.745) |
| rs4338381 | 1p21 | COL11A1 | N/A | G | 15 | 21153/24423/7119 | 0.599 | 0.431 | 0.23 (0.12, 0.33) | <0.001 | 44% (0.034) |
| rs4630309 | 11q13 | BBS1-CTSF | 0.975 | A | 21 | 35065/22326/3670 | 0.426 | 0.498 | 0.21 (0.10, 0.32) | <0.001 | 33% (0.075) |
| rs4752805 | 11p11 | PTPRJ | 1.000 | G | 21 | 34377/23095/3986 | 0.441 | 0.555 | 0.17 (0.06, 0.28) | 0.002 | 0% (0.957) |
| rs4864546 | 4q12 | CLOCK | N/A | A | 16 | 22000/25010/7089 | 0.593 | 0.519 | 0.09 (-0.01, 0.19) | 0.093 | 0% (0.930) |
| rs488133 | 6q25 | ESR1 | N/A | A | 16 | 5921/23878/24298 | 0.891 | 0.398 | 0.19 (0.08, 0.30) | <0.001 | 19% (0.235) |
| rs572169 | 3q26 | GHSR | 1.000 | A | 21 | 29323/26147/5973 | 0.523 | 0.596 | 0.37 (0.27, 0.47) | <0.001 | 0% (0.729) |
| rs611203 | 2q35 | PLCD4 | N/A | G | 16 | 9590/26226/18253 | 0.823 | 0.533 | 0.19 (0.09, 0.29) | <0.001 | 0% (0.820) |
| rs6180 | 5p13 | GHR | N/A | C | 16 | 11305/26766/16015 | 0.791 | 0.725 | 0.14 (0.04, 0.24) | 0.005 | 4% (0.410) |
| rs7087728 | 10q22 | MAT1A | N/A | A | 14 | 29812/12919/1471 | 0.326 | 0.222 | 0.18 (0.04, 0.32) | 0.01 | 0% (0.690) |
| rs709939 | 14q22 | SAMD4A | 0.882 | G | 20 | 11099/27670/16172 | 0.798 | 0.625 | 0.15 (0.05, 0.25) | 0.003 | 21% (0.200) |
| rs7137534 | 12p12 | PDE3A | N/A | A | 16 | 24393/23749/5979 | 0.549 | 0.457 | 0.25 (0.14, 0.35) | <0.001 | 48% (0.016) |
| rs7659604 | 4q27 | BBS7 | N/A | A | 16 | 18902/26131/9051 | 0.651 | 0.302 | 0.12 (0.02, 0.22) | 0.024 | 37% (0.068) |
| rs7751726 | 6p21 | PPARD | 0.931 | A | 20 | 55397/3499/80 | 0.061 | 0.469 | 0.13 (-0.15, 0.41) | 0.353 | 0% (0.801) |
| rs780094 | 2p23 | GCKR | 1.000 | A | 21 | 9606/29316/22523 | 0.844 | 0.614 | 0.27 (0.18, 0.37) | <0.001 | 0% (0.526) |
| rs7921 | 17q24 | GH1-GH2 | 1.000 | A | 21 | 33740/23570/4126 | 0.451 | 0.233 | 0.32 (0.21, 0.42) | <0.001 | 35% (0.059) |
| rs8071847 | 17p13 | POLR2A | 0.883 | G | 20 | 35780/18543/2572 | 0.371 | 0.487 | 0.17 (0.05, 0.29) | 0.005 | 0% (0.460) |
| rs8081612 | 17q23 | MAP3K3 | 0.972 | A | 20 | 25692/20056/4000 | 0.484 | 0.273 | 0.38 (0.26, 0.49) | <0.001 | 32% (0.083) |
| rs8108622 | 19p13 | INSR | 0.823 | A | 20 | 34329/19439/2908 | 0.394 | 0.331 | 0.09 (-0.03, 0.21) | 0.131 | 19% (0.221) |
| rs8111085 | 19p13 | ADAMTS10 | N/A | G | 16 | 47502/6361/249 | 0.122 | 0.235 | 0.17 (-0.03, 0.38) | 0.09 | 37% (0.071) |
| rs8115394 | 20q11 | MMP24 | 0.987 | G | 21 | 29967/25523/5591 | 0.509 | 0.562 | 0.38 (0.27, 0.48) | <0.001 | 0% (0.641) |
| rs864745 | 7p15 | JAZF1 | 1.000 | A | 21 | 15895/30570/14938 | 0.741 | 0.415 | 0.14 (0.05, 0.24) | 0.003 | 8% (0.361) |
| rs867529 | 2p12 | EIF2AK3 | N/A | G | 16 | 28119/21693/4289 | 0.48 | 0.548 | 0.24 (0.12, 0.35) | <0.001 | 9% (0.353) |
| rs867633 | 12q15 | HMGA2 | 0.970 | A | 21 | 9271/28906/22270 | 0.847 | 0.471 | 0.37 (0.27, 0.47) | <0.001 | 0% (0.722) |
| rs900 | 1q41 | TGFB2 | 1.000 | T | 21 | 32738/24178/4445 | 0.466 | 0.49 | 0.11 (0.00, 0.22) | 0.042 | 21% (0.192) |
| rs938609 | 15q26 | ACAN | 0.999 | A | 21 | 25335/28116/7962 | 0.587 | 0.436 | 0.15 (0.05, 0.25) | 0.003 | 15% (0.267) |
| rs959260 | 17q24 | GRB2 | N/A | G | 16 | 1763/15460/36869 | 0.967 | 0.348 | 0.38 (0.25, 0.51) | <0.001 | 12% (0.312) |
| rs9844666 | 3q21 | PCCB | 0.999 | A | 21 | 3612/22476/35307 | 0.941 | 0.503 | 0.20 (0.09, 0.32) | <0.001 | 0% (0.570) |
| rs9892365 | 17q23 | TBX2 | 0.997 | A | 21 | 27496/27180/6768 | 0.553 | 0.509 | 0.23 (0.13, 0.33) | <0.001 | 0% (0.673) |
|  |  |  |  |  |  |  |  |  |  |  |  |

*HWE denotes Hardy-Weinberg equilibrium.

**Heterogeneity across studies was quantified using I^2^ statistic. P values are from Cochran‘s Q test.

**TABLE S6.** SNPs used for the summary-level instrumental variable analysis. Data correspond to 180 SNPs reported from GIANT Consortium with corresponding CHD estimate reported in CARDIoGRAMplusC4D. Derived instrumental variable estimates are provided.

|  |  |  | GIANT Consortium | | CARDIoGRAMplusC4D | |  |  |
| --- | --- | --- | --- | --- | --- | --- | --- | --- |
| SNP (rs#) | Gene | Reference allele | Height beta coefficient | Height standard error | CHD log odds | CHD standard error | IV estimate log odds | IV estimate standard error |
| rs10010325 | TET2 | C | 0.024 | 0.004 | -0.002 | 0.010 | -0.085 | 0.396 |
| rs10037512 | MEF2C | C | 0.032 | 0.004 | 0.013 | 0.014 | 0.421 | 0.448 |
| rs1013209 | ADAM28 | C | -0.025 | 0.004 | 0.018 | 0.016 | -0.718 | 0.668 |
| rs10152591 | TLE3 | A | 0.041 | 0.006 | -0.002 | 0.015 | -0.054 | 0.366 |
| rs1043515 | PIP4K2B | A | -0.023 | 0.004 | 0.012 | 0.009 | -0.512 | 0.384 |
| rs1046934 | TSEN15 | C | -0.044 | 0.004 | -0.013 | 0.017 | 0.302 | 0.384 |
| rs1046943 | ZBTB24 | G | 0.020 | 0.004 | 0.009 | 0.014 | 0.474 | 0.711 |
| rs1047014 | ID4 | C | -0.032 | 0.004 | -0.018 | 0.021 | 0.565 | 0.650 |
| rs10748128 | FRS2 | G | 0.038 | 0.004 | -0.011 | 0.021 | -0.297 | 0.543 |
| rs10770705 | SLCO1C1 | C | 0.033 | 0.004 | -0.009 | 0.010 | -0.259 | 0.292 |
| rs10799445 | JMJD4 | C | 0.032 | 0.004 | 0.007 | 0.017 | 0.215 | 0.528 |
| rs10838801 | PTPRJ/SLC39A13 | G | -0.027 | 0.004 | 0.025 | 0.015 | -0.939 | 0.583 |
| rs10863936 | DTL | A | -0.021 | 0.003 | -0.003 | 0.009 | 0.153 | 0.409 |
| rs10874746 | RPL5 | T | -0.024 | 0.004 | 0.008 | 0.009 | -0.337 | 0.380 |
| rs11107116 | SOCS2 | G | 0.052 | 0.004 | 0.001 | 0.010 | 0.010 | 0.200 |
| rs11118346 | LYPLAL1 | C | -0.025 | 0.004 | 0.005 | 0.009 | -0.197 | 0.344 |
| rs11144688 | PCSK5 | A | -0.049 | 0.007 | 0.044 | 0.017 | -0.892 | 0.364 |
| rs11205277 | SF3B4 | G | -0.046 | 0.004 | -0.012 | 0.020 | 0.263 | 0.438 |
| rs11259936 | ADAMTSL3 | C | -0.044 | 0.004 | -0.005 | 0.009 | 0.118 | 0.200 |
| rs11599750 | CPN1 | T | -0.028 | 0.004 | 0.008 | 0.009 | -0.276 | 0.330 |
| rs11648796 | NARFL | G | -0.034 | 0.005 | 0.070 | 0.035 | -2.051 | 1.079 |
| rs11684404 | EIF2AK3 | C | -0.028 | 0.004 | -0.005 | 0.015 | 0.162 | 0.526 |
| rs1173727 | NPR3 | C | 0.034 | 0.004 | -0.033 | 0.014 | -0.970 | 0.428 |
| rs11830103 | SBNO1 | G | -0.035 | 0.004 | -0.012 | 0.017 | 0.348 | 0.484 |
| rs11867479 | KCNJ16/KCNJ2 | C | 0.025 | 0.004 | -0.008 | 0.009 | -0.339 | 0.371 |
| rs11958779 | SLC38A9 | G | -0.027 | 0.004 | 0.017 | 0.015 | -0.622 | 0.559 |
| rs12153391 | FBXW11 | A | -0.030 | 0.004 | 0.014 | 0.011 | -0.468 | 0.357 |
| rs12470505 | CCDC108/IHH | T | 0.041 | 0.006 | 0.008 | 0.015 | 0.184 | 0.359 |
| rs12474201 | SOCS5 | G | 0.028 | 0.004 | 0.011 | 0.014 | 0.381 | 0.520 |
| rs12534093 | IGF2BP3 | T | -0.034 | 0.004 | 0.002 | 0.017 | -0.057 | 0.512 |
| rs1257763 | PTPDC1 | A | 0.069 | 0.011 | -0.017 | 0.027 | -0.244 | 0.390 |
| rs12680655 | ZFAT | C | 0.028 | 0.004 | -0.018 | 0.014 | -0.636 | 0.510 |
| rs12694997 | SEPT2 | A | -0.024 | 0.004 | 0.005 | 0.011 | -0.194 | 0.458 |
| rs12902421 | MYO9A | C | -0.062 | 0.011 | 0.029 | 0.043 | -0.463 | 0.699 |
| rs12982744 | DOT1L | C | -0.030 | 0.004 | 0.023 | 0.015 | -0.773 | 0.503 |
| rs13088462 | DOCK3 | T | -0.052 | 0.008 | 0.002 | 0.020 | -0.044 | 0.391 |
| rs13177718 | FER | T | -0.040 | 0.007 | 0.011 | 0.017 | -0.271 | 0.426 |
| rs1325598 | PAPPA2 | G | -0.022 | 0.004 | 0.007 | 0.014 | -0.308 | 0.639 |
| rs1330 | NUCB2 | T | 0.022 | 0.004 | 0.007 | 0.009 | 0.305 | 0.422 |
| rs1351164 | TNS1 | C | 0.034 | 0.004 | 0.016 | 0.017 | 0.463 | 0.509 |
| rs1351394 | HMGA2 | C | 0.060 | 0.004 | -0.022 | 0.009 | -0.367 | 0.147 |
| rs143384 | GDF5 | G | -0.063 | 0.004 | -0.021 | 0.018 | 0.329 | 0.286 |
| rs1468758 | LPAR1 | T | -0.026 | 0.004 | 0.003 | 0.011 | -0.111 | 0.410 |
| rs1490384 | C6orf173 | C | 0.034 | 0.004 | -0.017 | 0.009 | -0.514 | 0.262 |
| rs1570106 | RAD51L1 | C | -0.026 | 0.005 | -0.030 | 0.017 | 1.136 | 0.699 |
| rs1582931 | CEP120 | G | -0.023 | 0.004 | 0.002 | 0.014 | -0.097 | 0.603 |
| rs1659127 | MKL2 | G | 0.027 | 0.004 | -0.039 | 0.021 | -1.461 | 0.822 |
| rs16942341 | ACAN | T | -0.130 | 0.012 | 0.026 | 0.028 | -0.196 | 0.220 |
| rs16964211 | CYP19A1 | A | -0.050 | 0.008 | 0.024 | 0.017 | -0.486 | 0.351 |
| rs17081935 | POLR2B | T | 0.030 | 0.005 | 0.044 | 0.011 | 1.460 | 0.418 |
| rs1708299 | JAZF1 | A | 0.040 | 0.004 | 0.021 | 0.010 | 0.534 | 0.260 |
| rs17318596 | ATP5SL | A | 0.032 | 0.004 | 0.039 | 0.010 | 1.220 | 0.355 |
| rs17346452 | DNM3 | C | -0.040 | 0.004 | 0.008 | 0.016 | -0.208 | 0.395 |
| rs1738475 | HTR1D | C | 0.025 | 0.004 | -0.016 | 0.014 | -0.636 | 0.571 |
| rs17391694 | GIPC2 | T | 0.042 | 0.006 | 0.010 | 0.016 | 0.245 | 0.377 |
| rs1741344 | SMOX | T | -0.023 | 0.004 | 0.022 | 0.009 | -0.956 | 0.429 |
| rs17511102 | CDC42EP3 | T | -0.060 | 0.007 | -0.005 | 0.025 | 0.081 | 0.416 |
| rs17780086 | LRRC37B | G | 0.028 | 0.005 | -0.013 | 0.019 | -0.458 | 0.691 |
| rs17782313 | MC4R | C | -0.028 | 0.004 | -0.026 | 0.011 | 0.935 | 0.404 |
| rs17806888 | SUCLG2 | C | 0.036 | 0.006 | -0.001 | 0.014 | -0.017 | 0.394 |
| rs1814175 | FOLH1 | C | 0.022 | 0.004 | 0.008 | 0.015 | 0.361 | 0.689 |
| rs1950500 | NFATC4 | C | 0.034 | 0.004 | -0.010 | 0.009 | -0.305 | 0.279 |
| rs2066807 | STAT2 | C | -0.054 | 0.007 | 0.031 | 0.018 | -0.578 | 0.349 |
| rs2072153 | ZNF652 | C | 0.021 | 0.004 | -0.025 | 0.015 | -1.209 | 0.742 |
| rs2079795 | TBX2 | C | 0.040 | 0.004 | -0.008 | 0.009 | -0.205 | 0.233 |
| rs2093210 | SIX6 | C | -0.032 | 0.004 | -0.002 | 0.015 | 0.068 | 0.483 |
| rs2110001 | TMEM176A | C | -0.031 | 0.004 | 0.001 | 0.019 | -0.044 | 0.611 |
| rs2145272 | BMP2 | G | -0.039 | 0.004 | -0.014 | 0.009 | 0.348 | 0.233 |
| rs2145998 | PPIF | T | -0.026 | 0.004 | 0.003 | 0.014 | -0.130 | 0.537 |
| rs2154319 | SCMH1 | C | -0.030 | 0.004 | 0.028 | 0.039 | -0.932 | 1.314 |
| rs2237886 | KCNQ1 | T | 0.046 | 0.006 | -0.019 | 0.015 | -0.404 | 0.322 |
| rs2247341 | SLBP/FGFR3 | G | 0.025 | 0.004 | -0.008 | 0.009 | -0.310 | 0.357 |
| rs2256183 | MICA | G | 0.040 | 0.004 | -0.026 | 0.018 | -0.643 | 0.463 |
| rs227724 | NOG | T | -0.030 | 0.004 | -0.033 | 0.019 | 1.091 | 0.642 |
| rs2279008 | MYO9B | C | 0.025 | 0.004 | -0.002 | 0.024 | -0.071 | 0.951 |
| rs2284746 | MFAP2 | C | -0.040 | 0.004 | 0.013 | 0.014 | -0.317 | 0.353 |
| rs2336725 | RTF1 | T | -0.027 | 0.004 | 0.006 | 0.009 | -0.215 | 0.338 |
| rs2341459 | C2orf34 | T | 0.025 | 0.004 | 0.003 | 0.010 | 0.121 | 0.396 |
| rs237743 | ZNFX1 | G | 0.041 | 0.004 | -0.032 | 0.017 | -0.788 | 0.422 |
| rs2580816 | NPPC | C | -0.045 | 0.005 | -0.017 | 0.019 | 0.379 | 0.423 |
| rs2597513 | HDAC11 | T | -0.036 | 0.006 | -0.002 | 0.015 | 0.061 | 0.406 |
| rs2629046 | SERPINE2 | C | 0.024 | 0.004 | -0.017 | 0.014 | -0.689 | 0.590 |
| rs2638953 | CCDC91 | C | 0.032 | 0.004 | 0.006 | 0.010 | 0.176 | 0.308 |
| rs2665838 | CSH1/GH1 | C | -0.042 | 0.004 | -0.016 | 0.016 | 0.386 | 0.379 |
| rs26868 | CASKIN1 | T | 0.034 | 0.004 | 0.001 | 0.020 | 0.035 | 0.602 |
| rs274546 | SLC22A5 | A | -0.029 | 0.004 | 0.018 | 0.009 | -0.629 | 0.312 |
| rs2778031 | SPIN1 | C | 0.031 | 0.004 | -0.013 | 0.016 | -0.415 | 0.532 |
| rs2780226 | HMGA1 | C | -0.076 | 0.007 | -0.048 | 0.017 | 0.632 | 0.234 |
| rs2834442 | KCNE2 | T | 0.026 | 0.004 | -0.035 | 0.014 | -1.360 | 0.585 |
| rs2856321 | ETV6 | G | -0.029 | 0.004 | -0.007 | 0.014 | 0.251 | 0.496 |
| rs2871865 | IGF1R | C | 0.057 | 0.006 | -0.035 | 0.024 | -0.616 | 0.420 |
| rs310405 | FAM46A | G | 0.026 | 0.004 | -0.011 | 0.009 | -0.427 | 0.342 |
| rs3110496 | ANKRD13B | G | -0.022 | 0.004 | 0.007 | 0.009 | -0.301 | 0.420 |
| rs3118905 | DLEU7 | G | -0.056 | 0.004 | -0.003 | 0.016 | 0.056 | 0.282 |
| rs3129109 | OR2J3 | C | -0.032 | 0.004 | 0.013 | 0.015 | -0.391 | 0.458 |
| rs3764419 | ATAD5/RNF135 | A | -0.035 | 0.004 | 0.013 | 0.009 | -0.382 | 0.255 |
| rs3782089 | SSSCA1 | C | -0.058 | 0.008 | -0.027 | 0.018 | 0.471 | 0.313 |
| rs3791675 | EFEMP1 | T | -0.053 | 0.004 | 0.020 | 0.010 | -0.385 | 0.192 |
| rs3812163 | BMP6 | T | -0.036 | 0.004 | 0.010 | 0.014 | -0.269 | 0.391 |
| rs4072910 | ADAMTS10 | C | -0.031 | 0.004 | 0.015 | 0.018 | -0.488 | 0.582 |
| rs42235 | CDK6 | C | 0.057 | 0.004 | -0.010 | 0.010 | -0.171 | 0.174 |
| rs422421 | FGFR4/NSD1 | C | -0.031 | 0.004 | -0.022 | 0.011 | 0.702 | 0.382 |
| rs425277 | PRKCZ | C | 0.022 | 0.004 | -0.015 | 0.010 | -0.674 | 0.455 |
| rs4282339 | SLIT3 | G | -0.036 | 0.004 | -0.009 | 0.011 | 0.239 | 0.294 |
| rs4470914 | TWISTNB | C | 0.029 | 0.005 | -0.048 | 0.019 | -1.639 | 0.693 |
| rs4601530 | CLIC4 | T | -0.028 | 0.004 | 0.042 | 0.018 | -1.493 | 0.674 |
| rs4605213 | NME2 | C | 0.021 | 0.004 | -0.015 | 0.015 | -0.714 | 0.709 |
| rs4640244 | KCNJ12 | G | 0.024 | 0.004 | -0.043 | 0.023 | -1.798 | 1.004 |
| rs4665736 | DNAJC27 | C | 0.029 | 0.004 | -0.006 | 0.014 | -0.201 | 0.493 |
| rs473902 | PTCH1/FANCC | T | 0.065 | 0.008 | 0.035 | 0.018 | 0.536 | 0.291 |
| rs4800452 | CABLES1 | C | 0.051 | 0.004 | 0.002 | 0.011 | 0.037 | 0.209 |
| rs4821083 | SYN3 | C | 0.031 | 0.005 | 0.012 | 0.018 | 0.401 | 0.587 |
| rs494459 | TREH | C | 0.020 | 0.004 | -0.013 | 0.009 | -0.642 | 0.459 |
| rs4965598 | ADAMTS17 | T | -0.028 | 0.004 | 0.014 | 0.011 | -0.513 | 0.388 |
| rs4986172 | ACBD4 | T | -0.032 | 0.004 | 0.011 | 0.009 | -0.358 | 0.294 |
| rs5017948 | OR4A5 | T | 0.027 | 0.005 | -0.020 | 0.019 | -0.737 | 0.699 |
| rs526896 | PITX1 | G | 0.030 | 0.004 | 0.000 | 0.010 | -0.014 | 0.332 |
| rs543650 | ESR1 | G | -0.034 | 0.004 | -0.035 | 0.020 | 1.041 | 0.604 |
| rs572169 | GHSR | C | 0.033 | 0.004 | -0.015 | 0.009 | -0.459 | 0.289 |
| rs5742915 | PML | C | -0.031 | 0.004 | 0.021 | 0.020 | -0.681 | 0.654 |
| rs634552 | SERPINH1 | G | 0.039 | 0.005 | -0.082 | 0.022 | -2.100 | 0.630 |
| rs6439167 | C3orf47 | C | -0.034 | 0.004 | 0.043 | 0.016 | -1.264 | 0.509 |
| rs6449353 | LCORL | C | 0.075 | 0.005 | -0.026 | 0.012 | -0.344 | 0.162 |
| rs6457620 | HLA locus | C | -0.029 | 0.004 | 0.024 | 0.014 | -0.833 | 0.487 |
| rs6457821 | PPARD/FANCE | C | -0.104 | 0.015 | 0.010 | 0.037 | -0.094 | 0.358 |
| rs6470764 | GSDMC | C | -0.050 | 0.005 | -0.015 | 0.010 | 0.302 | 0.210 |
| rs6473015 | PEX2 | A | -0.029 | 0.004 | 0.020 | 0.010 | -0.699 | 0.343 |
| rs654723 | FLI1 | A | 0.025 | 0.004 | 0.007 | 0.011 | 0.279 | 0.459 |
| rs6569648 | L3MBTL3 | T | -0.040 | 0.004 | 0.014 | 0.010 | -0.340 | 0.262 |
| rs6684205 | TGFB2 | G | -0.028 | 0.004 | -0.006 | 0.016 | 0.217 | 0.563 |
| rs6699417 | PKN2 | C | 0.021 | 0.004 | -0.028 | 0.009 | -1.352 | 0.484 |
| rs6714546 | LTBP1 | G | -0.026 | 0.004 | -0.023 | 0.020 | 0.883 | 0.789 |
| rs6879260 | GFPT2 | T | -0.022 | 0.004 | 0.017 | 0.009 | -0.794 | 0.424 |
| rs6959212 | STARD3NL | C | -0.024 | 0.004 | -0.003 | 0.009 | 0.144 | 0.386 |
| rs7027110 | ZNF462 | G | 0.031 | 0.004 | -0.007 | 0.010 | -0.237 | 0.327 |
| rs7112925 | RHOD | T | -0.023 | 0.004 | -0.002 | 0.009 | 0.081 | 0.394 |
| rs7155279 | TRIP11 | G | -0.024 | 0.004 | -0.013 | 0.014 | 0.544 | 0.603 |
| rs7178424 | C2CD4A | T | -0.021 | 0.004 | 0.014 | 0.009 | -0.645 | 0.430 |
| rs720390 | IGF2BP2 | A | 0.029 | 0.004 | 0.008 | 0.010 | 0.272 | 0.330 |
| rs724016 | ZBTB38 | A | -0.070 | 0.004 | 0.024 | 0.009 | -0.338 | 0.126 |
| rs7274811 | ZNF341 | G | -0.041 | 0.004 | -0.013 | 0.010 | 0.315 | 0.256 |
| rs7319045 | GPC5 | G | 0.025 | 0.004 | -0.022 | 0.014 | -0.862 | 0.586 |
| rs7332115 | PDS5B/BRCA2 | T | -0.023 | 0.004 | 0.017 | 0.011 | -0.751 | 0.474 |
| rs7460090 | SDR16C5 | C | 0.058 | 0.005 | -0.020 | 0.022 | -0.340 | 0.379 |
| rs7466269 | FUBP3 | A | 0.032 | 0.004 | 0.004 | 0.009 | 0.118 | 0.286 |
| rs7507204 | NFIC | C | 0.036 | 0.004 | 0.032 | 0.017 | 0.902 | 0.484 |
| rs751543 | PAPPA | T | 0.026 | 0.004 | 0.010 | 0.010 | 0.399 | 0.400 |
| rs7532866 | LIN28 | A | 0.021 | 0.004 | -0.008 | 0.009 | -0.375 | 0.440 |
| rs7567288 | NCKAP5 | C | -0.032 | 0.005 | -0.014 | 0.011 | 0.451 | 0.349 |
| rs7567851 | PDE11A | C | 0.037 | 0.007 | -0.032 | 0.028 | -0.858 | 0.784 |
| rs7689420 | HHIP | C | -0.073 | 0.005 | -0.009 | 0.012 | 0.130 | 0.163 |
| rs7697556 | ADAMTS3 | C | 0.028 | 0.004 | -0.010 | 0.009 | -0.349 | 0.317 |
| rs7759938 | LIN28B | C | -0.045 | 0.004 | 0.001 | 0.009 | -0.033 | 0.206 |
| rs7763064 | GPR126 | G | -0.048 | 0.004 | -0.005 | 0.010 | 0.104 | 0.198 |
| rs7849585 | QSOX2 | G | 0.029 | 0.004 | -0.012 | 0.016 | -0.422 | 0.544 |
| rs7853377 | C9orf64 | G | -0.024 | 0.004 | 0.006 | 0.017 | -0.231 | 0.707 |
| rs7864648 | BNC2 | G | 0.022 | 0.004 | -0.001 | 0.017 | -0.064 | 0.766 |
| rs788867 | PRKG2/BMP3 | G | -0.043 | 0.004 | -0.002 | 0.015 | 0.044 | 0.349 |
| rs7909670 | CCDC3 | C | -0.021 | 0.004 | 0.008 | 0.014 | -0.392 | 0.672 |
| rs7926971 | TEAD1 | G | -0.023 | 0.004 | -0.016 | 0.014 | 0.690 | 0.619 |
| rs7971536 | CCDC53/GNPTAB | T | -0.028 | 0.004 | 0.020 | 0.015 | -0.700 | 0.531 |
| rs798489 | GNA12 | C | -0.048 | 0.004 | -0.001 | 0.010 | 0.012 | 0.210 |
| rs8052560 | CTU2/GALNS | C | 0.029 | 0.005 | -0.007 | 0.028 | -0.229 | 0.980 |
| rs806794 | Histone cluster | G | 0.052 | 0.004 | 0.004 | 0.010 | 0.077 | 0.185 |
| rs8181166 | ZCCHC6 | C | 0.026 | 0.004 | -0.011 | 0.015 | -0.433 | 0.562 |
| rs822552 | PDIA4 | C | -0.025 | 0.004 | 0.007 | 0.011 | -0.270 | 0.448 |
| rs862034 | LTBP2 | G | -0.028 | 0.004 | -0.014 | 0.014 | 0.488 | 0.515 |
| rs889014 | BOD1 | T | -0.030 | 0.004 | 0.004 | 0.009 | -0.121 | 0.312 |
| rs891088 | INSR | G | -0.029 | 0.004 | -0.013 | 0.010 | 0.440 | 0.349 |
| rs9360921 | SENP6 | G | -0.042 | 0.006 | -0.017 | 0.023 | 0.403 | 0.549 |
| rs9428104 | SPAG17 | G | -0.041 | 0.004 | 0.002 | 0.016 | -0.060 | 0.388 |
| rs9456307 | TULP4 | T | -0.048 | 0.008 | -0.024 | 0.031 | 0.504 | 0.658 |
| rs9472414 | SUPT3H/RUNX2 | T | -0.026 | 0.004 | 0.020 | 0.017 | -0.775 | 0.671 |
| rs955748 | WWC2 | A | -0.023 | 0.004 | 0.004 | 0.010 | -0.189 | 0.432 |
| rs961764 | VGLL2 | C | -0.024 | 0.004 | -0.028 | 0.014 | 1.179 | 0.611 |
| rs9835332 | C3orf63 | C | -0.026 | 0.004 | 0.000 | 0.014 | -0.003 | 0.539 |
| rs9844666 | PCCB | A | -0.024 | 0.004 | 0.033 | 0.010 | -1.361 | 0.471 |
| rs9863706 | RYBP | C | -0.031 | 0.004 | 0.005 | 0.017 | -0.146 | 0.538 |
| rs9967417 | DYM | C | -0.038 | 0.004 | -0.002 | 0.014 | 0.057 | 0.377 |
| rs9969804 | IPPK | A | 0.030 | 0.004 | -0.004 | 0.009 | -0.124 | 0.292 |

**TABLE S7. Sensitivity analysis to investigate the effect of weighting allele scores on the pooled summary estimates**

|  |  | **Allele score weighed by the summary beta coefficients  from the gene-centric meta-analysis** | | |  | **Allele score not weighted** | | |
| --- | --- | --- | --- | --- | --- | --- | --- | --- |
|  |  |  |  |  |  |  |  |  |
| **Cardiovascular endpoints:** |  | *Odds ratio for a 6.5cm increase in height  (95% CI)* | *P value,  Z-test* | *Heterogeneity, I^2^ (Cochran’s Q test P-value)* |  | *Odds ratio for a 6.5cm increase in height (95% CI)* | *P value,  Z-test* | *Heterogeneity, I^2^ (Cochran’s Q test P-value)* |
|  |  |  |  |  |  |  |  |  |
| Coronary heart disease |  | 0.90 (0.78 to 1.03) | 0.110 | 38% (0.050) |  | 0.89 (0.77 to 1.02) | 0.082 | 41% (0.033) |
| Stroke |  | 0.97 (0.79 to 1.19) | 0.779 | 0% (0.547) |  | 0.99 (0.81 to 1.23) | 0.951 | 0% (0.749) |
|  |  |  |  |  |  |  |  |  |
|  |  |  |  |  |  |  |  |  |
| **Cardiovascular risk factors:** |  | *Difference per SD in trait for a 6.5cm increase in height  (95% CI)* | *P value,  Z-test* | *Heterogeneity, I^2^ (Cochran’s Q test P-value)* |  | *Difference per SD in trait for a 6.5cm increase in height (95% CI)* | *P value,  Z-test* | *Heterogeneity, I^2^ (Cochran’s Q test P-value)* |
|  |  |  |  |  |  |  |  |  |
| Body mass index |  | -0.10 (-0.15 to -0.05) | <0.001 | 30% (0.100) |  | -0.09 (-0.14 to -0.04) | <0.001 | 32% (0.083) |
| Systolic blood pressure |  | -0.05 (-0.10 to 0.00) | 0.064 | 0% (0.467) |  | -0.04 (-0.09 to 0.01) | 0.102 | 0% (0.793) |
| Triglycerides* |  | -0.10 (-0.16 to -0.05) | <0.001 | 0% (0.513) |  | -0.13 (-0.19 to -0.07) | <0.001 | 1% (0.438) |
| HDL cholesterol |  | 0.02 (-0.03 to 0.08) | 0.432 | 33% (0.089) |  | 0.04 (-0.02 to 0.10) | 0.210 | 29% (0.119) |
| Non-HDL cholesterol |  | -0.12 (-0.17 to -0.06) | <0.001 | 32% (0.102) |  | -0.12 (-0.18 to -0.06) | <0.001 | 36% (0.067) |
| FEV_1_ |  | 0.26 (0.15 to 0.36) | <0.001 | 0% (0.735) |  | 0.28 (0.17 to 0.39) | <0.001 | 0% (0.892) |
| FVC |  | 0.30 (0.20 to 0.41) | <0.001 | 0% (0.774) |  | 0.33 (0.22 to 0.43) | <0.001 | 0% (0.835) |
| Fasting glucose |  | -0.04 (-0.10 to 0.01) | 0.127 | 13% (0.302) |  | -0.04 (-0.10 to 0.02) | 0.162 | 0% (0.563) |
| C-reactive protein* |  | -0.07 (-0.13 to 0.00) | 0.042 | 0% (0.761) |  | -0.09 (-0.16 to -0.03) | 0.005 | 0% (0.619) |
|  |  |  |  |  |  |  |  |  |

Presented are odds ratios or differences per standard deviation (SD) unit for different cardiovascular endpoints and CV risk factors per 6.5 cm increase in adult height. An odds ratio<1 or a difference<0 indicates that the cardiovascular disease risk decreases with increased adult height. Heterogeneity across studies is shown using I^2^ (%) and P values from χ^2^ tests for heterogeneity. *Effects of triglycerides and C-reactive protein are estimated after log-transformation. FEV_1_: forced expiratory volume in 1 second; FVC: forced vital capacity; HDL: high-density lipoprotein.

**Figure S1. Forest plot showing the study-specific and pooled IV estimates of height on risk of CHD. Studies are sorted by standard error. Estimates represent a 1-SD (6.5cm) increase in height**

**
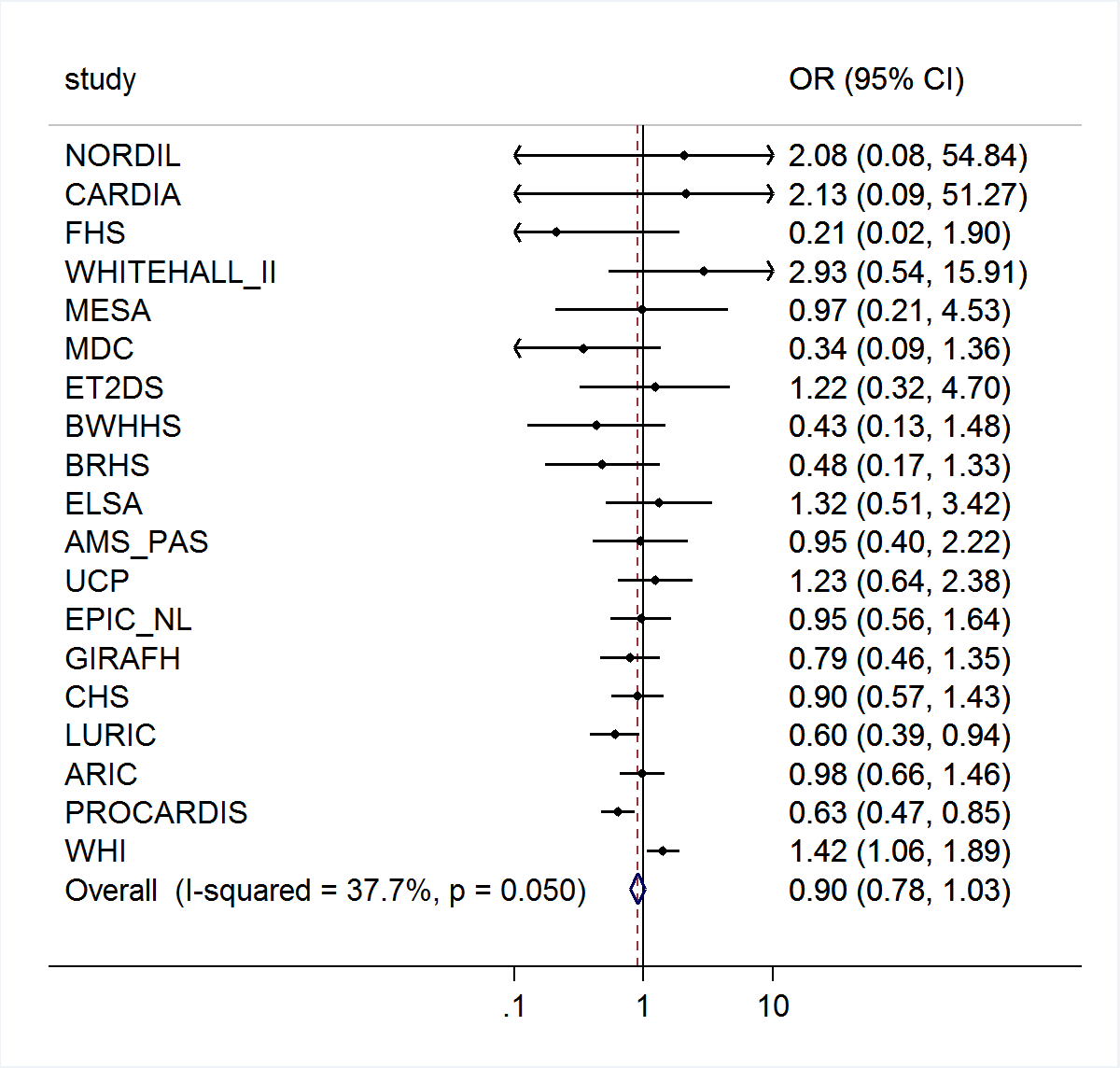
**

**Figure S2. Forest plot showing the study-specific and pooled IV estimates of height on risk of stroke. Studies are sorted by standard error. Estimates represent a 1-SD (6.5cm) increase in height**

**
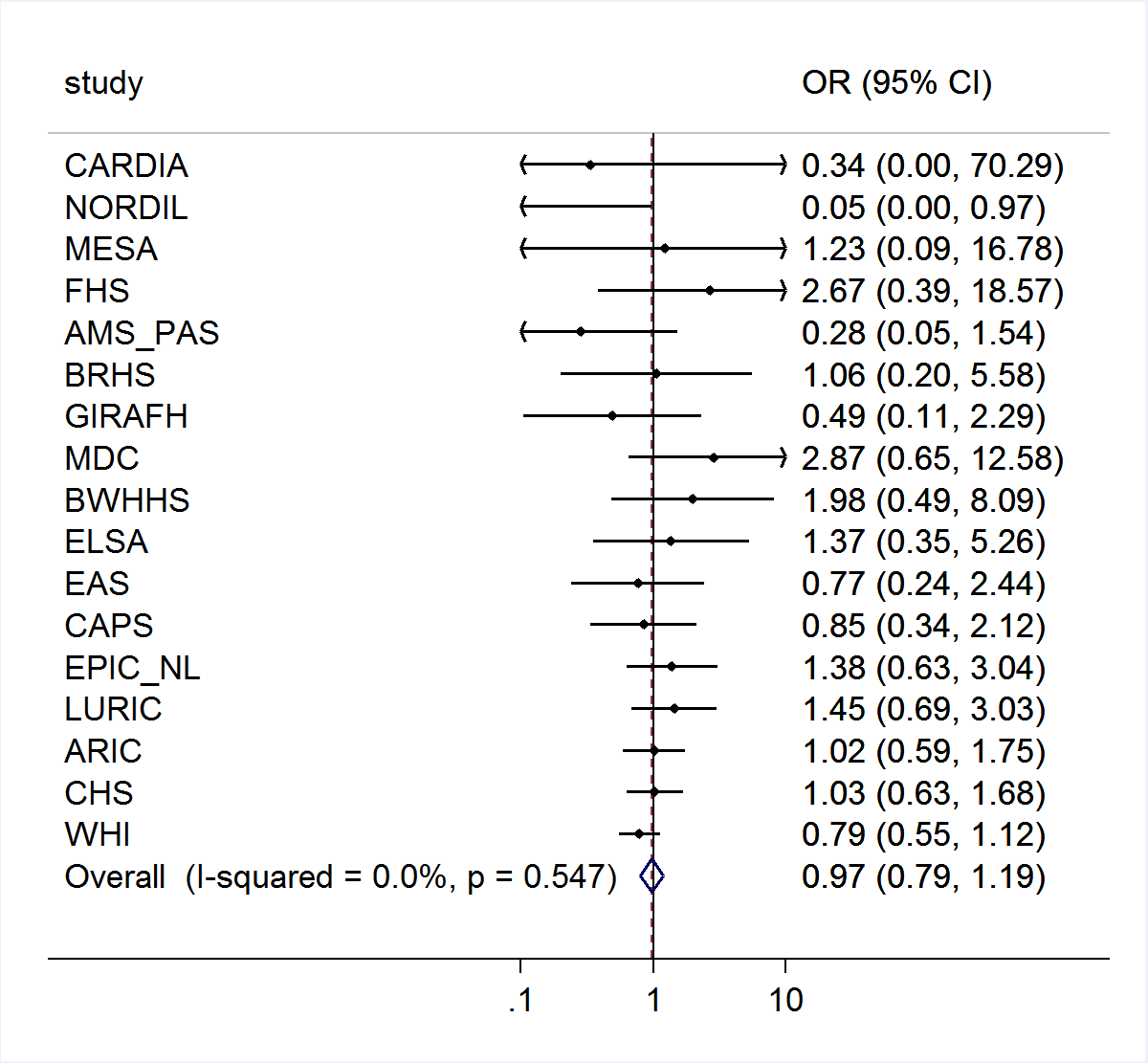
**

**Figure S3. Forest plot of the instrumental variable estimates for CHD per SNP, modelled to a 1-SD (6.5cm) increase in height.**

**
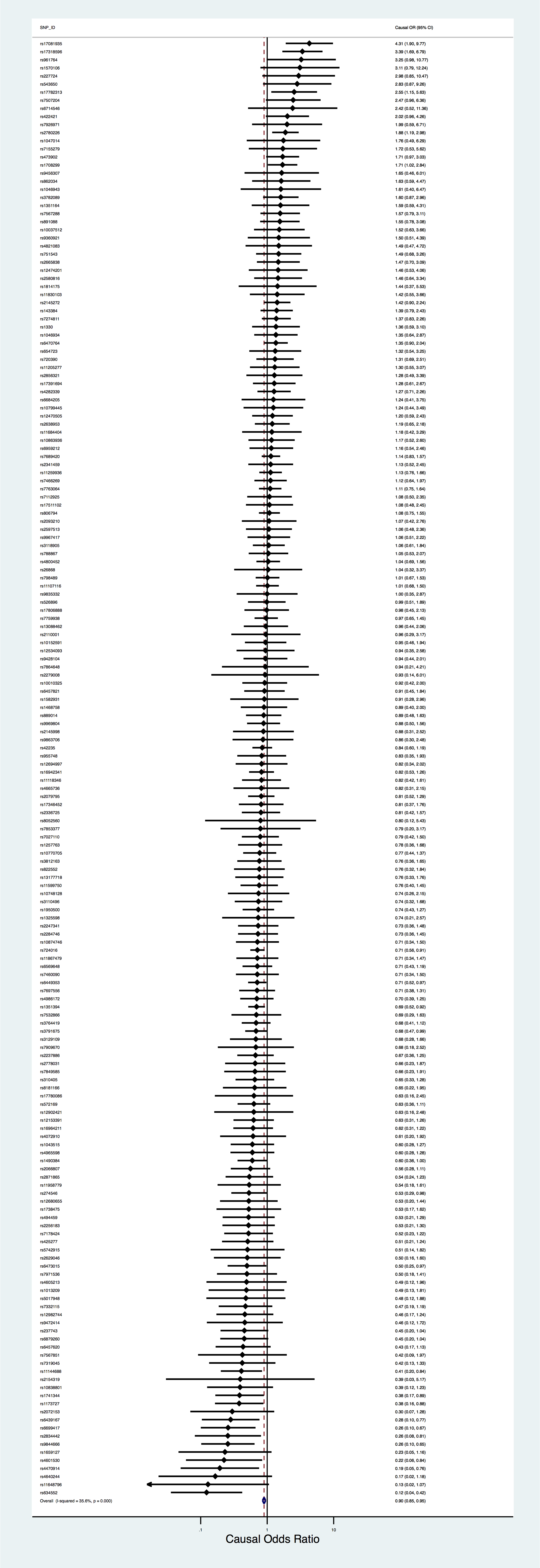
**

**Figure S4. Cross-hair plot to show the association of SNPs with difference in height and CHD risk**

**FIGURE S5. Meta-analysis pooled causal effects for a 6.5cm increase in height on cardiovascular events adjusted for cardiovascular traits.**

| **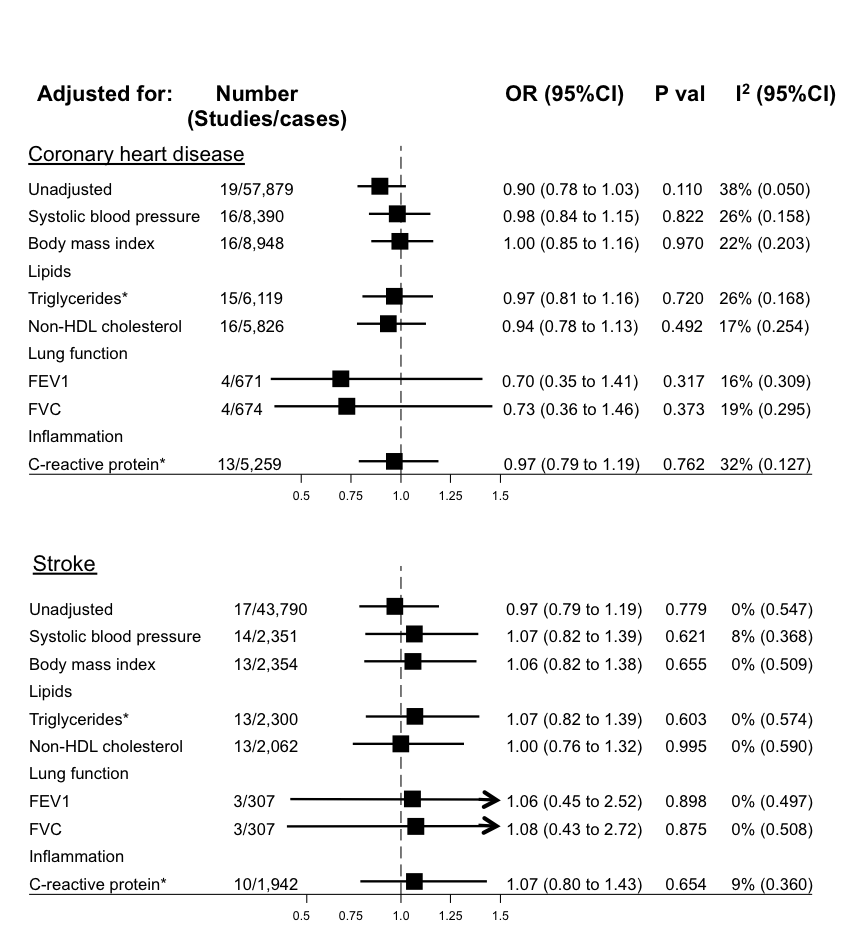** |
| --- |

Pooled causal effects between cardiovascular disease outcomes and adult height adjusted for different cardiovascular traits. Causal odds ratios and corresponding 95% confidence intervals (CI) are estimated from fixed-effect meta-analysis of estimates from instrumental variable analyses in individual studies. Heterogeneity across studies is shown using I^2^ (%) and P values from χ^2^ tests for heterogeneity. *Effects of triglycerides and C-reactive protein are estimated after log-transformation.
